# GRASP: a response-locked paradigm that reveals low-beta desynchronisation is a participant-invariant signature of semantic decoding in EEG

**DOI:** 10.3389/fnhum.2026.1913105

**Published:** 2026-09-14

**Authors:** Moein Radman, Joshua James Podmore, Riccardo Poli, Silke Paulmann, Ian Daly

**Affiliations:** 1 Brain-Computer Interfaces and Neural Engineering Laboratory, School of Computer Science and Electronic Engineering, University of Essex, Colchester, United Kingdom; 2 Department of Psychology, University of Essex, Colchester, United Kingdom

**Keywords:** EEG, low-beta desynchronisation, pseudo-word baseline, response-locked decoding, semantic brain-computer interface, semantic retrieval, threshold-free cluster enhancement, time-frequency analysis

## Abstract

Conventional Brain-Computer Interfaces (BCIs) typically rely on sequential selection mechanisms, limiting communication speed and increasing dependence on preserved sensory or motor pathways. Semantic BCIs (sBCIs) aim to overcome this limitation by decoding concepts directly from neural activity. Owing to the continuous nature of semantic processing, progress in this domain requires a principled characterisation of the electrophysiological contrast between a so-called high-semantic state (meaningful concept retrieval) and low-semantic state (pseudo-word recognition) in scalp EEG. Here, we introduce GRASP (Game of Retrieval and Semantic Processing), a response-locked auditory paradigm involving the identification of concrete concepts from spoken sentence definitions. Our clues relate to three distinct semantic categories: body parts, foods, and musical instruments. Phonologically matched pseudo-word dummy trials preserved sentence-like auditory structure while substantially reducing recoverable lexical-semantic content, providing a controlled low-semantic reference condition. Participants were instructed to press a spacebar when they felt reasonably confident in their answer, focusing the EEG analysis on the pre-response retrieval interval. Data were captured from 13 neurologically healthy British English speakers. Preprocessed 64-channel EEG epochs were analysed using Morlet time-frequency decomposition over 2–35 Hz. Concept-minus-dummy contrast maps were computed per participant for each category and entered into a whole-scalp three-dimensional Threshold-Free Cluster Enhancement (TFCE)-based permutation analysis across channel × frequency × time space. All three categories produced significant TFCE-corrected effects in the pre-response interval, consistently located in the low-beta range and peaking at approximately 15 Hz. Meaningful concept trials showed lower low-beta power than dummy trials, with complete directional consistency across all 13 participants in all three contrasts. Body and music trial effects were strongest over centro-parietal and posterior scalp regions, whereas food showed a broader frontal-central-posterior distribution. These findings identify a robust, participant-invariant low-beta desynchronisation signature distinguishing meaningful semantic retrieval from a controlled pseudo-word baseline. The consistency of this effect across categories and participants suggests that the high-semantic *vs*. low-semantic boundary may provide a more cross-participant-stable electrophysiological target than fine-grained concept *vs*. concept discrimination, providing a principled signal-characterisation framework for future sBCI development.

## 1 Introduction

### 1.1 Clinical motivation

Semantic brain-computer interfaces (sBCIs) aim to decode entire concepts from neural activity in a single operation without exogenous stimulation or residual motor output (Rybář et al., 2021; Rybář and Daly, 2022). If realised, such systems would be transformative for individuals in a locked-in state owing to conditions such as Amyotrophic Lateral Sclerosis (ALS) (Linse et al., 2018) or profound congenital impairments such as Cerebral Palsy (CP) (Pennington et al., 2004), providing a communication channel grounded solely in ideation. Existing communication-oriented BCI paradigms typically rely on signals that require intact sensory or motor pathways. For example, visual-P300 and Steady State Visual Evoked Potential (SSVEP) speller systems depend on reliable gaze control and sustained visual fixation (Sutton et al., 1965; Farwell and Donchin, 1988; Salvaris et al., 2012; Li et al., 2020; Poli et al., 2025; Regan, 1966; Chen et al., 2015; Norcia et al., 2015). In contrast, Motor Imagery (MI) BCIs do not depend on peripheral motor output but require extensive calibration and training periods that are difficult to sustain across the trajectory of progressive motor neuron disease (Pfurtscheller and Aranibar, 1977; Chaudhary et al., 2016; Abiri et al., 2019). As the disease advances and each exogenous modality becomes less reliable, a system operating entirely through endogenous ideation becomes correspondingly more relevant. sBCIs are well positioned to accommodate these populations, however realising the potential utility of this control signal requires understanding both the neural architecture that semantic processing exploits and whether its electrophysiological signature is sufficiently robust and generalisable to support cross-participant decoding.

### 1.2 The neural architecture of semantic processing

The hub-and-spoke model of semantic cognition proposes that conceptual knowledge is supported by a network of modality-specific spoke regions encoding sensory, motor, and linguistic properties, which converge at an amodal hub in the bilateral Anterior Temporal Lobes (ATL) (Patterson et al., 2007; Lambon Ralph et al., 2017). Two converging lines of neuropsychological evidence establish the bilateral implementation of the hub. Bilateral ATL atrophy in semantic dementia produces severe, cross-modal and cross-category degradation of conceptual knowledge (Patterson et al., 2007; Lambon Ralph et al., 2017). Unilateral ATL resection for temporal lobe epilepsy produces only mild, test-sensitive impairment because each hemisphere can partially sustain hub function when the other is compromised (Lambon Ralph et al., 2012; Rouse et al., 2024a; Rouse et al., 2024b). Huth et al. (2016) provided large-scale empirical support for the distributed spoke system by mapping representations for over 10,000 words across the cortical surface during continuous narrative listening, finding that semantic content is continuously and bilaterally distributed with no sharp categorical boundaries between regions. Crucially, this topological continuity is not incidental. The semantic space is not divisible into orthogonal groups as lateral connections even between the most frequently utilised categories of animals *vs.* tools exist, such as needle *vs.* snake, mouse *vs.* computer, hammer *vs.* shark. This shared semantic space introduces complications when assigning data with discrete labels, however, fMRI evidence demonstrates that semantic representations are decodable from neural activity and can be generalised to concepts not seen during classifier training (Mitchell et al., 2008). In other words, the relational and associative nature of concepts allows for the accurate definition of activation projections for novel concepts based on the signal properties of a known subset.

Further, early EEG work established above-chance single-trial semantic decoding within and across participants (Murphy et al., 2011). Crucially, the presence of participant invariant signals provides key evidence for the basic feasibility of sBCI, showing models should not require unfeasible feature extraction, classifier training, and recalibration schemes. Despite this, previous research discussed thus far often involved the direct exogenous presentation of stimuli during data capture. Efforts to remove these perceptual confounds via delayed ideation paradigms proved successful for fNIRS but failed to translate to EEG platforms (Rybář et al., 2021; Rybář et al., 2024). The introduction of word-guessing paradigms subtly avoided these perceptual confounds by never explicitly cueing target concept words. Again, these showed significant promise in an fMRI setting (Kivisaari et al., 2019), however, they largely failed during translation to electrophysiological data capture (magnetoencephalography) (Ghazaryan et al., 2023). Our key adaptation to this methodology, namely a response-locked motor proxy (spacebar press) at the point of concept retrieval (the “eureka” moment), substantively increased the relative quality of the associated epochs by removing redundant post-retrieval trial samples, allowing for the single-participant binary decoding of concept *vs.* concept trials (Podmore et al., 2026).

Despite this, our classification performance is still modest (approx. 70% accuracy). This may reflect a structural problem in how the comparison is framed. Note that concept *vs.* concept classification targets graded representational differences between categories, but the hub-and-spoke architecture predicts that these differences will be topographically continuous and idiosyncratic across individuals, because each individual’s conceptual representations are grounded in their own unique sensorimotor history. Our coordinate-based fMRI meta-analysis across 75 studies confirmed that category-specific activation patterns are distinct but substantially overlapping (Radman et al., 2025), and analysis of decoding methodology demonstrates that standard categorical classification is poorly equipped to detect the conjoint, distributed neural codes that neurocomputational models of semantic cognition predict (Frisby et al., 2023). What may vary less across participants than the graded differences between categories is the contrast between meaningful concept retrieval and pseudo-word processing with substantially reduced recoverable lexical-semantic content. Whether lexical-semantic content is being recovered at all is a more cross-participant-stable contrast than which concept is being recovered. The fundamental practical barrier for sBCI is therefore not whether semantic signals are present in neural data, but whether they can be isolated against a principled low-semantic reference that renders the contrast more participant-invariant.

### 1.3 Signal characterisation as a developmental prerequisite

Signal characterisation against such a reference condition is the prerequisite for all downstream methodological development. Without knowing the spatial, temporal, and spectral properties of the contrast between a so-called high-semantic and controlled low-semantic processing in scalp EEG, any classifier architecture or preprocessing choice remains undirected. The developmental trajectory of the Steady-State Evoked Potential (SSVEP) field illustrates what focused characterisation enables. Once the harmonic structure of the SSVEP response was established, canonical correlation analysis could be borrowed from multivariate statistics and refined into filter-bank architectures capable of real-world information transfer rates well in excess of 100 bpm (Regan, 1966; Norcia et al., 2015; Lin et al., 2006; Chen et al., 2015). Semantic signals differ fundamentally from SSVEPs in terms of their endogenous origin and latency-variability, so the developmental trajectory will not be identical, but the principle holds. In the simplest terms, characterisation precedes optimisation, and a comparable starting point for sBCI requires a principled reference condition against which the spatio-spectro-temporal properties of the semantic signal set can be mapped.

### 1.4 The semantic processing continuum

Defining an adequate low-semantic reference condition is not straightforward, and this difficulty may go some way toward explaining the absence of this experimental feature in the literature. Resting-state baselines are the obvious candidate but are not adequate, as these low-level baselines have been found to activate the same regions as semantic tasks, plausibly reflecting ongoing conceptual processing during undirected, idle thought (Visser et al., 2010). Mind-wandering during such low-demand periods recruits the same default-network regions active at rest and increases as task demand falls (Mason et al., 2007), while functioning as a goal-driven process directed at content other than the task at hand rather than as an absence of cognition (Smallwood and Schooler, 2006). This kind of blurring between rest and task more broadly undermines treating resting state as a clean, content-free baseline (Northoff et al., 2023). Non-semantic cognitive tasks such as mental arithmetic present a structurally cleaner contrast but are similarly inadequate, since number concepts engage quantity semantics and recruit working-memory representations with verbal and linguistic components, producing an activation profile that is not interpretable as a specific, controlled position on the semantic continuum. Diffuse manipulations such as fatigue, sustained attention depletion, or divided attention would also reduce semantic processing to some degree, but act across multiple processing stages simultaneously and degrade auditory, attentional, and motor signals alongside any semantic component, leaving no means of isolating lexical-semantic integration specifically. Taken together, an effective semantic processing paradigm must reliably position participants at distal locations along the semantic continuum without introducing unrelated, degraded or undirected cognitive activity. Within this context, a low-semantic state requires minimal lexical-semantic processing, and a high-semantic state requires elevated lexical-semantic processing.

### 1.5 A neural precedent and its limitations

The Event-Related Potential (ERP) literature has long established that pseudo-word substitution suppresses the N400, the canonical marker of lexical-semantic integration, while leaving syntactic processing intact (Hahne and Jescheniak, 2001). Because pseudo-words are phonologically valid and embedded in grammatically well-formed sentences, low-level auditory processing and task demands are held constant by the paradigm design. This demonstrates that a gibberish word condition manipulation creates a controlled low-semantic reference condition at a specific and empirically characterised point in the processing stream. Here, the N400 establishes pseudo-words as a valid semantic null at the level of individual word processing. The degree to which this extends across the full representational geometry of ongoing sentence comprehension was addressed directly by Desbordes et al. (2023). This enhanced the traditional analyses from the study of averaged ERP components to an investigation of the dimensionality of the representational space as a whole. Using MEG and intracranial EEG, they showed that meaningful sentences elicit substantially higher representational dimensionality than jabberwocky (gibberish) sentences in which all content words are replaced by phonologically valid pseudo-words while syntactic structure, prosody, and task demands are preserved. They found three distinct dynamic patterns separating meaningful from jabberwocky sentence processing, consistent with sequential stages of lexical access, multiword integration, and sentence-level wrap-up.

This work demonstrates that meaningful and pseudo-word conditions produce measurably different representational dimensionality in electrophysiology, suggesting that N400 suppression by pseudo-words reflects a genuine collapse of representational depth rather than an isolated component effect. However, several features of the design limit direct transfer to an sBCI context. Most critically, the study used participants with intracranial electrodes implanted for the surgical management of epilepsy, rather than participants assessable via whole-brain non-invasive scalp recordings. Further, the analysis was conducted within participants using temporal generalisation rather than cross-participant characterisation of participant-invariant patterns, which any deployable system requires. Beyond these primary barriers, the stimuli were visual, presented word by word in rapid serial visual presentation, whereas auditory sentence definitions avoid reliance on intact reading ability and visual fixation, both of which may be compromised in the target clinical populations described above. Finally, the contrast was characterised globally across a general vocabulary without targeting specific concept categories, leaving open whether the dimensional signature of the pseudo-word contrast is consistent across concepts with different modal grounding.

### 1.6 The present study: GRASP

Here we introduce GRASP (Game of Retrieval and Semantic Processing) and report a cross-participant spatio-spectro-temporal characterisation of its concept *vs.* dummy (pseudo-word) contrasts across the three semantic categories of body parts, musical instruments, and food concepts during a word guessing game paradigm. These categories were selected based on our fMRI semantic concept meta-analyses (Radman et al., 2025). The categories were noted as the most highly separable of those assessed. Body parts are grounded in somatosensory and motor systems, instruments in auditory-motor systems, and food in visceral-limbic systems. These differences should prove informative when comparing the respective dummy *vs.* concept trials, as the lower relative degree of overlap may reveal patterns that are invariant across categories at the hub level and divergent in the distributed periphery, as the hub-and-spoke architecture predicts.

Crucially, GRASP addresses the perceptual cue-epoch confound identified by Rybář et al. (2024), in which cue-locked analysis conflates exogenous sensory responses with endogenous conceptual processing and overestimates apparent semantic decoding performance. We achieve this here via a response-locked evidence-accumulation design. All trials are epoched to the spacebar press, which serves as a motor proxy for the word identification event. Note that we target the pre-response window during which the concept is being actively retrieved. The paradigm replaces orthographic clues with spoken sentence definitions, routing processing through auditory cortical networks (Marinkovic et al., 2003). Evidence from concurrent MEG and EEG confirms that the ATL hub is engaged by compositional sentence-level semantics as well as single-word meaning (Law et al., 2026), supporting the expectation that spoken sentence definitions will engage the hub throughout the pre-response window.

The defining feature of our GRASP platform is a within-session pseudo-word condition in which one quarter of trials present acoustically matched sentences with all content words replaced by phonologically valid nonsense words. As the dummy condition provides a controlled state with substantially reduced recoverable lexical-semantic content, its contrast with meaningful concept retrieval may be more participant-invariant than graded differences between concept categories. Where Desbordes et al. (2023) characterised the semantic *vs.* non-semantic contrast in MEG and intracranial EEG within participants and without concept specificity, GRASP uses scalp EEG and applies a cluster-based spatio-spectral permutation analysis to characterise this contrast separately for each of the three concept categories across participants, directly addressing the translational gaps that require clarity for sBCI development. We evaluate whether the controlled contrast exposes invariant spatio-spectro-temporal features across participants and investigate if those features differ across concept categories in ways consistent with the hub-and-spoke architecture. Given that scalp EEG offers limited spatial resolution and lacks the cross-participant co-registration available to fMRI, the spatial patterns we report should be interpreted as approximate electrode-space topographies rather than precise source localisations. We hope that these findings will provide the signal characterisation necessary to begin directed development of scalp EEG-based sBCI.

### 1.7 Hypotheses

#### 1.7.1 Hypothesis 1: bilateral anterior temporal hub signature

On the basis of the hub-and-spoke model (Patterson et al., 2007; Lambon Ralph et al., 2017) and the bilateral redundancy evidence (Lambon Ralph et al., 2012), we predict that a primary locus of discrimination between dummy and concept trials will be centred on bilateral anterior temporal regions, regardless of concept category, with additional recruitment of inferior frontal cortex as the semantic control component of the broader Controlled Semantic Cognition framework (Lambon Ralph et al., 2017). The bilateral ATL constitutes the amodal semantic hub in the hub-and-spoke architecture, and any reliable contrast between high-semantic and controlled low-semantic conditions should engage this hub systematically. The bilateral rather than strictly left-lateralised prediction reflects both the Huth et al. (2016) continuous bilateral maps and the redundancy evidence showing the right ATL can compensate for the left in unilateral damage (Lambon Ralph et al., 2012).

#### 1.7.2 Hypothesis 2: category-sensitive topographic divergence

Beyond this common hub and on the basis of the spatial distribution characterised in our fMRI meta-analysis (Radman et al., 2025), we predict that the topographic profiles of dummy *vs.* concept discrimination will diverge across categories in ways that reflect their modal grounding. Body part concepts are predicted to show lateralised differences in occipito-parietal and somatosensory regions, consistent with the occipito-parietal network identified in the meta-analysis and with the embodied language literature establishing somatotopic motor and premotor representation of action words associated with different body effectors (Pulvermüller, 2005; Hauk et al., 2004). Musical instrument concepts are predicted to show a bilateral signature over superior temporal and insular regions, reflecting the auditory-motor architecture documented for music processing, which identified convergent activations in bilateral superior temporal gyrus, insula, and precentral gyrus (Radman et al., 2025). Food concepts are the most constrained prediction. The meta-analysis identified food-related activations predominantly in limbic and subcortical structures, including parahippocampal gyrus, insula, putamen, and claustrum, many of which are deep and poorly resolved by scalp EEG. Accordingly, any observable food-related effects over scalp electrodes likely reflect secondary frontal or insular projections rather than primary source regions identified by fMRI.

## 2 Materials and methods

### 2.1 Overview of the analysis pipeline

The analysis pipeline is summarised in Figure 1. EEG data were acquired while participants completed the GRASP semantic retrieval task, which included meaningful concept trials and phonologically matched dummy trials (pseudo-words). Following preprocessing, cleaned response-locked EEG epochs were analysed using a two-stage time-frequency framework. First, participant-level time-frequency representations were computed for each condition and used to derive within-participant concept *vs.* dummy contrast maps. Second, these participant-level contrast maps were entered into a group-level non-parametric statistical analysis across the complete channel × frequency × time space.

**Figure 1:**
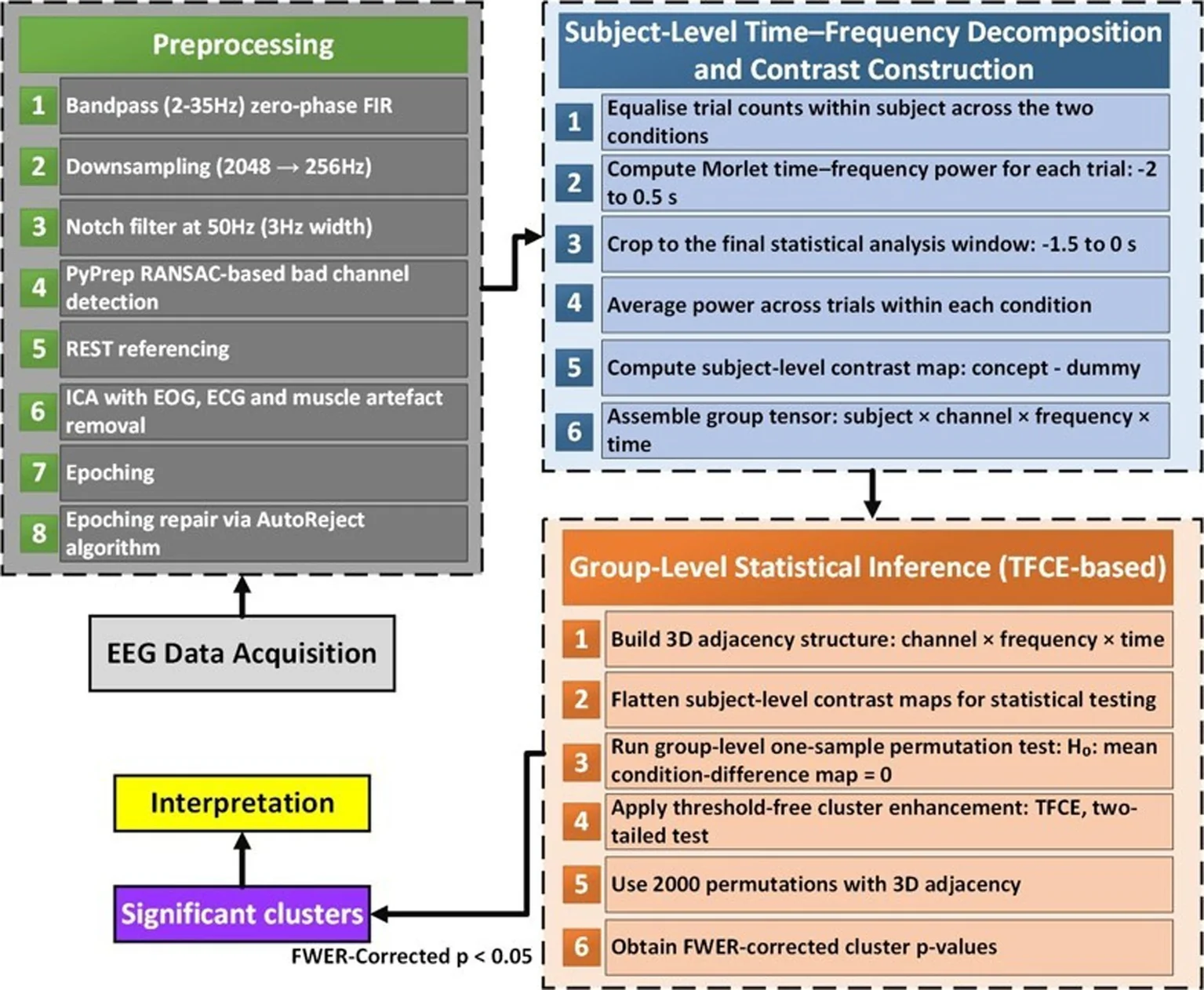
Overview of the response-locked whole-scalp time-frequency contrast analysis and TFCE-based group-level inference pipeline.

This pipeline was designed to characterise oscillatory activity associated with meaningful concept processing relative to dummy trials during the response-preparatory interval. Group-level inference was performed using a whole-scalp three-dimensional TFCE-based permutation approach (Smith and Nichols, 2009), allowing effects to be detected as coherent spatio-spectro-temporal patterns while controlling the family-wise error rate across sensors, frequencies, and time points. The main stages of the analysis were therefore EEG data acquisition, preprocessing, time-frequency decomposition, participant-level contrast construction, group-level TFCE inference, and characterisation of significant effects.

### 2.2 EEG data acquisition

#### 2.2.1 Participants

Thirteen neurologically healthy adults participated (8 Female, avg.: 23 years, range: 19–42). All participant recruitment was limited exclusively to British English speakers due to the extensive use of culturally specific phrases, idioms and associations present in the clue set stimuli. All participants provided written informed consent. Ethical approval was obtained from the University of Essex Ethics Committee, Ethics Sub Committee 1, under ERAMS reference ETH2425-0148.

#### 2.2.2 Stimulus generation

The same three semantic groupings from our Graded-Inventory of Semantic Triggers (GIST) paradigm were retained (Podmore et al., 2026): food items (9 fruits and vegetables), musical instruments (9 items), and body parts (9 items), yielding 27 concept items in total (see Figure 2). Each item sentence comprised a sequence of sub-clues that progressively increased in specificity toward the target item. A corresponding pseudo-word sentence variant was generated for each definition. For example, one meaningful clue sentence was: “This can be strong or weak, an opportunity can slip through these, and they can be printed for identification” (target item: fingers). The corresponding pseudo-word variant was: “This can be gronst or deak, an ordincunity can spip through these, and they can be predged for ufectolisation.” In the pseudo-word version, content words were replaced with phonologically matched pseudo-words, whereas function words, including auxiliaries, prepositions, modals, pronouns, and conjunctions, were retained. This procedure was intended to preserve the overall syntactic structure and broad phonological and auditory characteristics of the sentence while substantially reducing recoverable lexical-semantic content in the dummy condition (Figure 3).

**Figure 2:**
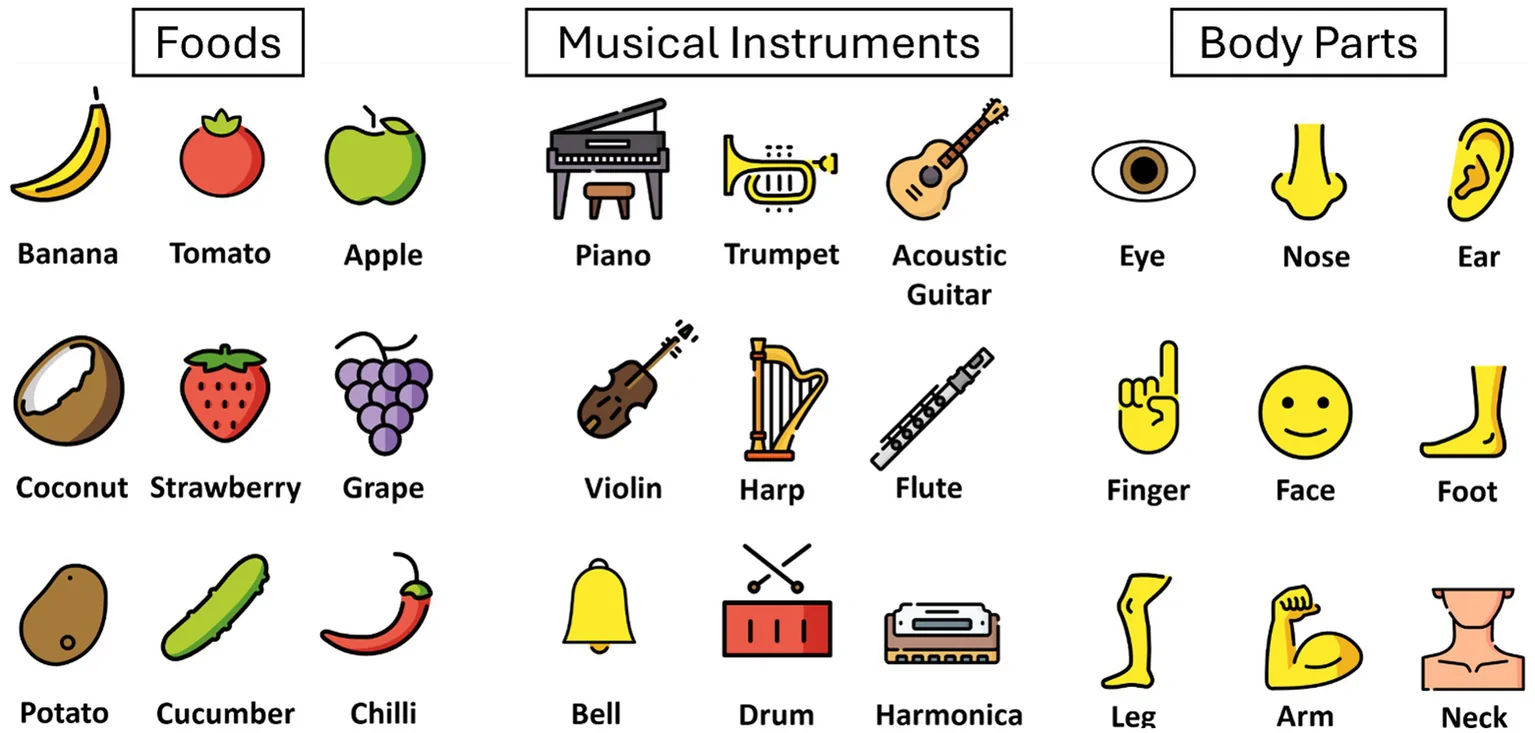
A pictographic of the 27 items which comprise the GRASP clue set. The three categories include: Foods (fruits/vegetables), musical instruments, and body parts. Emoji artwork reproduced from OpenMoji (Groß et al., 2026), licensed under CC BY-SA 4.0.

**Figure 3:**
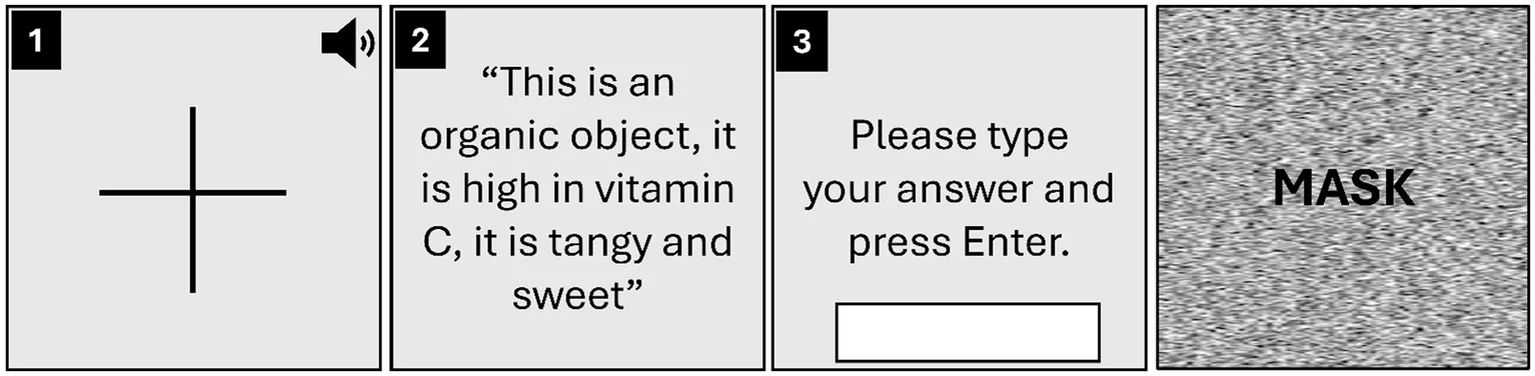
Schematic of the trial sequence. The speaker icon on the first (fixation cross) panel indicates audio onset and is included here for clarity only. The second panel is shown for illustrative purposes and does not appear during the experiment. Clue sentences are presented exclusively in auditory form and are never displayed as text. The third panel depicts the response screen participants see either when the audio finishes or immediately upon pressing the spacebar. The final narrow panel shows the dynamic Gaussian mask that appears at the end of each trial. Note that the word MASK is only presented here for illustrative purposes.

The pseudo-words were generated in two stages. Initially, the Wuggy library (Keuleers and Brysbaert, 2010) pseudo-word generator was employed (classic mode) with constraints applied for syllabic overlap. This was chosen over a simplistic letter-length matching protocol to ensure TTS duration and phonological similarity. Further, each candidate was iteratively validated for syllable count via the syllapy library (Holtzscher, 2024), and the pseudo-words were confirmed as having non-word status via dictionary lookup. Words without any viable generated variants were handled manually and verified by applying the same criteria.

The audio files were synthesised using Microsoft Edge TTS (voice: en-GB-RyanNeural) (Microsoft Corporation, 2024) in mono 22 KHz 16-bit WAV format. All word-level temporal bounds were computed as TextGrid files using the Montreal Forced Aligner (McAuliffe et al., 2017) employing an English (UK) pronunciation dictionary and acoustic model. Here, each experimental session comprised 12 blocks. These featured 36 trials each, with 27 standard and 9 pseudo-trials. Note that of the 324 pseudo-sentences generated, only a third (108) were selected to be presented in the finalised version of the clue set. This was done to ensure class balance with the other concept trials and to maintain consistency across participants. Crucially, each clue sentence is unique. This was enforced to encourage the participants to process each clue sentence fully and avoid participants simply recalling the terms associated with the target item clues.

#### 2.2.3 Experimental paradigm

The experiment was coded in PsychoPy (Peirce et al., 2019), with audio delivered via PyGame (Shinners, 2011). Twelve blocks of 36 trials were presented per session, with a total duration of approximately 3 hrs, including setup and rest periods. Participants were seated in front of a computer monitor and keyboard (height adjusted for comfort).

At the start of each block, a pictographic display presented images of all 27 target items for 5 s to orient participants to the active item set. For each trial, a fixation cross was presented at screen centre, immediately following this, a given clue sentence WAV audio file was played through a set of centrally positioned computer speakers. Participants were trained and instructed to press the spacebar once they felt they were approximately 95% confident in their answer. Participants were capable of interrupting the playback at any point to indicate early identification of the ground truth item. At this instant, both the onset of the spacebar press and the most recently presented word, as indexed via the MFA TextGrid info, were logged. Following either the audio playback completion or the interruption event, the fixation cross is removed, and a text input prompt is then shown onscreen, during which time participants were given 30 s to type their guess to the clue before a trial timeout protocol was initiated. On pseudo-word trials, the same instruction was provided, whereby participants were trained to perform the spacebar press once 95% confident that the trial belonged to the pseudo-word condition. Note that for these trials, participants were also required to type the word “dummy” and press enter to confirm attentive listening and ensure parity in the motor behaviour of the standard and pseudo-trials.

After the participant provided their answer and pressed enter, a 1.5 s dynamic Gaussian mask was presented, followed by a jittered blank pause (0.5–1.25 s). Once the block was completed, all responses for the 27 standard trials were processed using the SymSpell auto-correct algorithm (Garbe, 2012) via an item-word ranked weighting combined with WordNet lemmatisation (Miller, 1995). The cosine similarity between word2vec (Mikolov et al., 2013) embeddings of the corrected response and the corresponding ground-truth item label was computed to determine the trial-level semantic accuracy scores. Note that for dummy trials, the latency from the first pseudo-word onset to the spacebar response was calculated to ensure task engagement.

### 2.3 Preprocessing

Continuous scalp EEG was acquired at 2,048 Hz using a 64-channel BioSemi ActiveTwo system arranged in the standard 10–20 configuration, supplemented by four bipolar EOG channels (EXG1-4). All offline preprocessing was performed in MNE-Python (Gramfort et al., 2013). Raw data were first processed with a zero-phase bandpass FIR filter spanning 2–35 Hz. Data were then downsampled to 256 Hz and recording segments falling outside block boundaries were removed to retain only task-relevant data. A 50 Hz notch filter was then applied to attenuate mains interference. Following this, bad channels were identified using the RANSAC algorithm within PyPrep (Bigdely-Shamlo et al., 2015) and reconstructed by spherical spline interpolation (Perrin et al., 1989). Subsequently, continuous data were re-referenced to an approximate infinity reference using the REST framework (Yao, 2001; Dong et al., 2017).

Before ICA, segments containing transient artefacts and high-frequency muscle activity were annotated and masked from the fitting data to protect the quality of the decomposition. Extended Infomax ICA was applied with 32 components and 10,000 iterations (Makeig et al., 1996; Delorme and Makeig, 2004). Artefact-laden components were identified by computing z-score correlations between each component time course and the EOG and ECG (T7) reference channels, flagging those that exceeded a threshold of *z* = 2.0. After component removal, a final check identified and interpolated any residual bad channels.

Trials were subsequently screened by a behavioural quality filter, retaining only those with exact matches between the typed response and the target label. Additional quality controls were in place based on typing speed computed at the single-participant level. Here, typing duration was defined from first-to-last keypress, with an upper retention bound set at median + 4 × Median Absolute Deviation (MAD). In sum, adaptive flags filtered trials based on response-time validity, a backspace ratio ≤ 0.10, and a response-target cosine overlap ≥ 0.70. A final adaptive cleaning stage utilises the AutoReject algorithm (Jas et al., 2017) to maximise data retention by learning participant-specific thresholds and interpolating noisy channels within individual epochs rather than simply discarding them.

### 2.4 Participant-level time-frequency decomposition

Participant-level time-frequency decomposition was performed separately for each participant and condition using the cleaned response-locked EEG epochs. For each planned comparison, trial counts were matched within each participant across the two conditions before spectral estimation. This procedure removes surplus epochs from the condition with more available trials so that both conditions contribute the same number of epochs to the participant-level contrast. This ensured that condition-specific power estimates were derived from matched numbers of trials and reduced the possibility that differences between conditions reflected unequal averaging reliability.

Time-frequency power was estimated using Morlet wavelet decomposition over the 2–35 Hz range (Cohen, 2014). For each analysed frequency, the number of wavelet cycles was defined as the maximum between 3 and the frequency divided by 2.5, i.e.: $n_{\text{cycles}}=\max(3,\frac{f}{2.5})$ .

This setting provided a balance between temporal and spectral resolution across the analysed frequency range.

Wavelet decomposition was computed using a padded temporal support window from −2.0 to 0.5 s to reduce edge-related artefacts. Power values were then transformed using a log10 transformation. The resulting maps were cropped to the final response-locked statistical window from −1.5 to 0 s. No baseline correction was applied, and subsequent inference was based on within-participant condition differences in log10-transformed power. Because the contrast was computed as log10 (power_condition1) − log10(power_condition2), the resulting values are equivalent to log10 power ratios between conditions, rather than baseline-corrected dB values.

### 2.5 Participant-level contrast construction

For each participant and condition, single-trial power estimates were averaged across trials, producing one channel × frequency × time map per condition. For each participant, concept-related activity was quantified by subtracting dummy-trial power from concept-trial power: concept – dummy.

The concept condition corresponded to the semantic category tested in each comparison, such as food, body, or music. This produced one contrast map per participant for each planned comparison. Positive values in the contrast map indicate greater log-transformed power for concept trials than dummy trials, whereas negative values indicate lower power for concept trials relative to dummy trials. Because the subtraction was performed within each participant, the contrast reduced the influence of between-participant differences in overall EEG power and focused the analysis on condition-specific modulation.

The resulting maps were arranged into a four-dimensional tensor with dimensions: participant **×** channel × frequency × time. This tensor formed the input to the group-level statistical analysis, with participants treated as the unit of inference.

### 2.6 Group-level statistical inference

Group-level statistical inference was performed using a whole-scalp, three-dimensional non-parametric permutation framework with TFCE (Smith and Nichols, 2009; Maris and Oostenveld, 2007). This analysis aimed to identify reliable concept *vs.* dummy effects across the full spatio-spectro-temporal EEG feature space, while controlling for multiple comparisons across scalp channels, frequency bins, and time points.

For each planned comparison, the participant-level contrast maps were arranged into a four-dimensional tensor with dimensions participant × channel × frequency × time. Before statistical testing, the data structure was checked for consistency across participants. All included participants were required to have the same channel order, frequency vector, time vector, and contrast-map dimensionality. This ensured that each channel-frequency-time sample represented the same measurement location across participants.

Group-level inference was based on the participant-level condition-difference maps, with participants treated as the unit of analysis. For each channel-frequency-time sample, the concept *vs.* dummy contrast values were tested against zero using a one-sample permutation test. The null hypothesis was that the mean contrast across participants was equal to zero: H₀: mean (concept − dummy) = 0.

This corresponds to a random-effects group analysis, because inference was based on between-participant variability rather than trial-level variability. A two-tailed test was used to allow detection of both positive and negative concept *vs.* dummy effects. Positive effects indicate greater log-transformed time-frequency power for concept trials than dummy trials, whereas negative effects indicate lower power for concept trials relative to dummy trials.

To account for the structured nature of EEG time-frequency data, a three-dimensional adjacency structure was constructed across the full analysis space. Sensor adjacency was derived from the EEG channel montage, such that neighbouring scalp electrodes were treated as spatially adjacent. Frequency adjacency was defined between neighbouring frequency bins, and temporal adjacency was defined between neighbouring time samples. These three components were combined into a single channel × frequency × time adjacency matrix. This adjacency structure allowed the statistical procedure to evaluate effects as coherent spatio-spectro-temporal patterns rather than as isolated independent samples.

Multiple comparisons were controlled using TFCE-based permutation inference. In our analysis, no fixed cluster-forming threshold was selected. Instead, TFCE was applied with enhancement starting at zero and evaluated over successive statistic levels using a step size of 0.05. The TFCE score at each sample reflected both the local magnitude of the statistical effect and the degree of support from neighbouring samples in the combined channel × frequency × time space. Thus, effects were enhanced when they were both statistically strong and embedded within spatially, spectrally, or temporally coherent neighbourhoods. This made the analysis sensitive to distributed EEG effects that extended across neighbouring sensors, frequencies, and time points, while avoiding the need to define *a priori* electrodes, frequency bands, or latency windows.

Statistical significance was assessed using 2000 permutations. For each permutation, the maximum TFCE-enhanced statistic across the complete channel × frequency × time search space was retained to form a null distribution of maximum statistics. The observed TFCE-enhanced statistics were then compared against this permutation-derived null distribution. This maximum-statistic procedure provided family-wise error rate (FWER) control across all tested sensors, frequencies, and time points (Maris and Oostenveld, 2007). Effects were considered statistically significant at FWER-corrected *p* < 0.05. The resulting significant mask identified channel-frequency-time samples belonging to corrected spatio-spectro-temporal effects. These corrected effects were subsequently characterised in terms of their scalp distribution, frequency profile, and temporal evolution within the response-locked analysis window.

### 2.7 Peak-window selection and exploratory source-space projection

To provide an exploratory anatomical summary of the sensor-level TFCE effects, source-space projections were computed for a compact time-frequency window selected separately for each concept *vs.* dummy contrast. This analysis was performed after the whole-scalp TFCE inference and was not used to define statistical significance. Instead, the source-space maps were intended to visualise the approximate cortical distribution of the peak sensor-level effect.

For each contrast, the TFCE export contained the corrected significance mask, the observed group-level t-statistic image, the group mean contrast, and the corresponding channel, frequency, and time vectors. A peak-selection score was computed over the frequency × time plane by collapsing corrected statistical evidence across channels:


$$S(f,t)=\sum_{c}|T_{\mathrm{obs}}(c,f,t)|\times I_{\mathrm{sig}}(c,f,t)$$

Where $T_{\mathrm{obs}}(c,f,t)$ is the observed group-level statistic at channel c , frequency f , and time t , and $I_{\mathrm{sig}}(c,f,t)$ is the TFCE-corrected binary significance mask. This score therefore quantified the total corrected statistical evidence at each frequency–time bin, combining the magnitude and spatial extent of the significant effect while ignoring samples outside the corrected mask.

This peak-selection strategy was used to define a data-driven but statistically constrained summary window for source-space projection. Because TFCE inference was performed over the full channel × frequency × time space, selecting the source-projection window from a single electrode, a predefined frequency band, or an uncorrected effect map could have biased the anatomical summary toward an arbitrary sensor or descriptive maximum. Collapsing corrected evidence across channels instead identified the frequency–time point at which the corrected group-level effect was strongest at the scalp level, while restricting the selection to samples surviving FWER-corrected TFCE inference.

The selected peak was defined as the frequency–time bin with the maximum value of S(f,t) . A compact neighbourhood around this peak was then defined using a half-width of 3 Hz in frequency and 150 ms in time. This peak-centred neighbourhood was used only to summarise the already-estimated time–frequency effect for source-space projection and diagnostic visualisation. Note that it did not determine the spectral or temporal resolution of the Morlet decomposition itself and was not treated as an additional inferential test.

Source-space projection was performed using a linearly constrained minimum variance (LCMV) beamformer (Van Veen et al., 1997), which estimates activity at predefined source locations while suppressing contributions from elsewhere. The analysis was intended to provide a descriptive, window-specific projection of the corrected sensor-level power contrast rather than a full-brain statistical source localisation. The source model used the fsaverage template anatomy, an ico-5 cortical source space, and an EEG forward model (Dale et al., 1999), thereby constraining estimates to discrete template cortical vertices rather than participant-specific anatomy.

A single forward solution was used for all concept *vs*. dummy contrasts. LCMV filters were computed from the common covariance matrix using 0.05 regularisation, max-power orientation selection, unit-noise-gain normalisation, and reduced-rank estimation. These filters were then applied separately to the concept and dummy covariance matrices to estimate source power within the selected peak time–frequency window.

For each participant, the source-space contrast was computed as:


$$\log_{10}(\;\frac{P_{\text{concept}}+\varepsilon }{P_{\text{dummy}}+\varepsilon })$$

Where $P_{\text{concept}}$ and $P_{\text{dummy}}$ denote the LCMV source-power estimates for the concept and dummy conditions, respectively, and $\varepsilon =10^{-10}$ was added for numerical stability. This contrast is equivalent to the difference between log10-transformed source-power estimates for the two conditions. Participant-level source-contrast maps were averaged to obtain a group-level source projection for each contrast. These source-space maps were displayed on *fsaverage* pial anatomy as unthresholded descriptive visualisations.

Because individual structural MRIs were not available and source reconstruction was performed on a template anatomy using scalp EEG only, these projections were treated as exploratory anatomical summaries rather than precise source-localisation results. Statistical inference was therefore based on the whole-scalp TFCE analysis described above; the LCMV source maps were used only to visualise the approximate cortical distribution of the peak corrected time-frequency effects (Dale et al., 1999).

## 3 Results

All subsequent analyses were performed using epochs with a − 1.5 to 0 s window against the response-locked (spacebar press) event. These bounds were selected on the basis of a formal contamination analysis conducted across all spacebar-locked epochs from the 13 participants assessed. Contamination was defined as the proportion of windows extending before the current trial’s initiation trigger. At t_min = −2.0 s, 3.76% of spacebar epochs included inter-trial interval activity, at −1.5 s, this fell to 0.17%, and at −1.0 s, contamination was 0.00%. Based on these findings, −1.5 to 0 s epoching bounds represent a principled balance between retaining within-trial pre-response EEG samples and minimising cross-trial contamination. To reduce wavelet edge artefacts, all Morlet decompositions were computed on a padded window extending from −2.0 to +0.5 s and subsequent power maps were then cropped to the −1.5 to 0 s analysis window prior to statistical testing.

### 3.1 Behavioural performance

Behavioural performance was summarised across all recorded trials before the application of EEG-based or epoch-level behavioural quality filtering. Eleven participants completed all 432 planned trials, while participants 3 and 10 completed 396 trials because one block was not recorded for each owing to fatigue. Concept-identification accuracy was defined as the proportion of trials for which the participant’s typed response matched the target item label following response correction, while cosine similarity provided a less restrictive measure of response similarity. Response latency was defined as the interval between trial-audio onset and the spacebar press and was calculated only for trials in which a spacebar response occurred.

Across the 13 participants, mean exact-match accuracy for concept trials was 79.5% (SD = 7.5%), and mean cosine similarity between corrected responses and target labels was 89.0% (SD = 4.5%). Dummy responses were typed correctly in 100% of cases. Mean concept-trial response latency was 6.28 s (SD = 0.48), with a mean participant-level median of 6.33 s (SD = 0.51). For reference, the complete clue audio had a mean duration of 7.68 s (SD = 0.83). Dummy trials were identified earlier, with a mean response latency of 2.84 s (SD = 0.51) and a mean participant-level median of 2.62 s (SD = 0.51).

Performance also differed across semantic categories. Body trials produced the highest concept-identification accuracy, followed by food and music trials. Music trials showed both the longest concept response latency and the lowest accuracy, indicating that this was the most difficult category overall. Full descriptive behavioural results across the overall, Body, Food, and Music conditions are provided in Supplementary Table S1. Trial-by-trial confidence ratings were not collected; instead, participants were instructed to press the spacebar only once they felt approximately 95% confident in their judgement. The spacebar response should therefore be interpreted as an operational marker of the instructed confidence threshold rather than as an independent quantitative confidence measure.

### 3.2 Group-level TFCE-corrected results

Group-level TFCE-corrected time-frequency inference was performed for the three planned concept *vs.* dummy contrasts: Body *vs*. Dummy, Food *vs*. Dummy, and Music *vs*. Dummy. Table 1 provides the primary numerical summary of the corrected effects. Significant samples occupied 18.38% of the tested channel × frequency × time space for Body *vs*. Dummy, 12.28% for Food *vs*. Dummy, and 14.99% for Music *vs*. Dummy. The strongest corrected statistical evidence occurred before the response in all three contrasts, with peak times at −0.586 s, −0.852 s, and −0.762 s, respectively. The corresponding peak frequencies were consistently in the low-beta range: 14.51 Hz for Body − Dummy, 14.41 Hz for Food − Dummy, and 15.84 Hz for Music − Dummy. All contrasts showed negative concept-minus-dummy effects and complete participant-level directional consistency, with 13/13 participants matching the group-level direction in each contrast. Results for each contrast are presented as a triplet comprising the whole-scalp TFCE summary, the peak-selection diagnostic, and the exploratory LCMV source projection, shown for Body *vs*. Dummy in Figures 4–6, Food *vs*. Dummy in Figures 7–9, and Music *vs*. Dummy in Figures 10–12.

**Table 1:** TFCE-corrected concept *vs.* dummy effects across semantic categories.

Contrast	Significant samples (%)	Peak time (s)	Peak frequency (Hz)	Peak band	Top channels in peak window	Direction	Direction consistency	Directional consistency *p*
Body - Dummy	18.38%	−0.586	14.51	low beta	CP1, CP3, P1, PO7, P3, PO3, O1, C3, P7, P5	body < dummy	13/13 (100.0%)	< 0.001
Food - Dummy	12.28%	−0.852	14.41	low beta	CP1, C3, CP3, AF4, O1, AF8, F4, P1, FT8, C1	food < dummy	13/13 (100.0%)	< 0.001
Music - Dummy	14.99%	−0.762	15.84	low beta	C3, P3, CP3, P5, P1, FC3, CP5, PO7, P7, PO3	music < dummy	13/13 (100.0%)	< 0.001

Summary of TFCE-corrected concept *vs*. dummy effects. Significant samples indicate the percentage of tested channel × frequency × time samples surviving TFCE correction at FWER-corrected *p* < 0.05. Peak time and peak frequency were derived from corrected statistical summaries collapsed across the remaining dimensions. Top channels indicate the 10 highest-ranked electrodes within the corrected effect. Channel strength was defined as the mean absolute observed T statistic across significant frequency × time samples at that channel, i.e., the sum of |T_obs| over TFCE-corrected samples divided by the number of significant samples at that channel. Only samples surviving TFCE correction at FWER-corrected *p* < 0.05 contributed to this ranking. Direction is reported as concept − dummy. Directional consistency indicates the number and percentage of participants whose participant-level effect matched the group-level direction. Directional consistency p-values were computed using a one-sided binomial sign-consistency test. Table-level peaks summarise time-wise and frequency-wise corrected evidence after collapsing across the other dimensions. The source-localisation windows reported below were selected separately from the two-dimensional frequency × time corrected evidence map and therefore need not have identical peak times.

**Figure 4:**
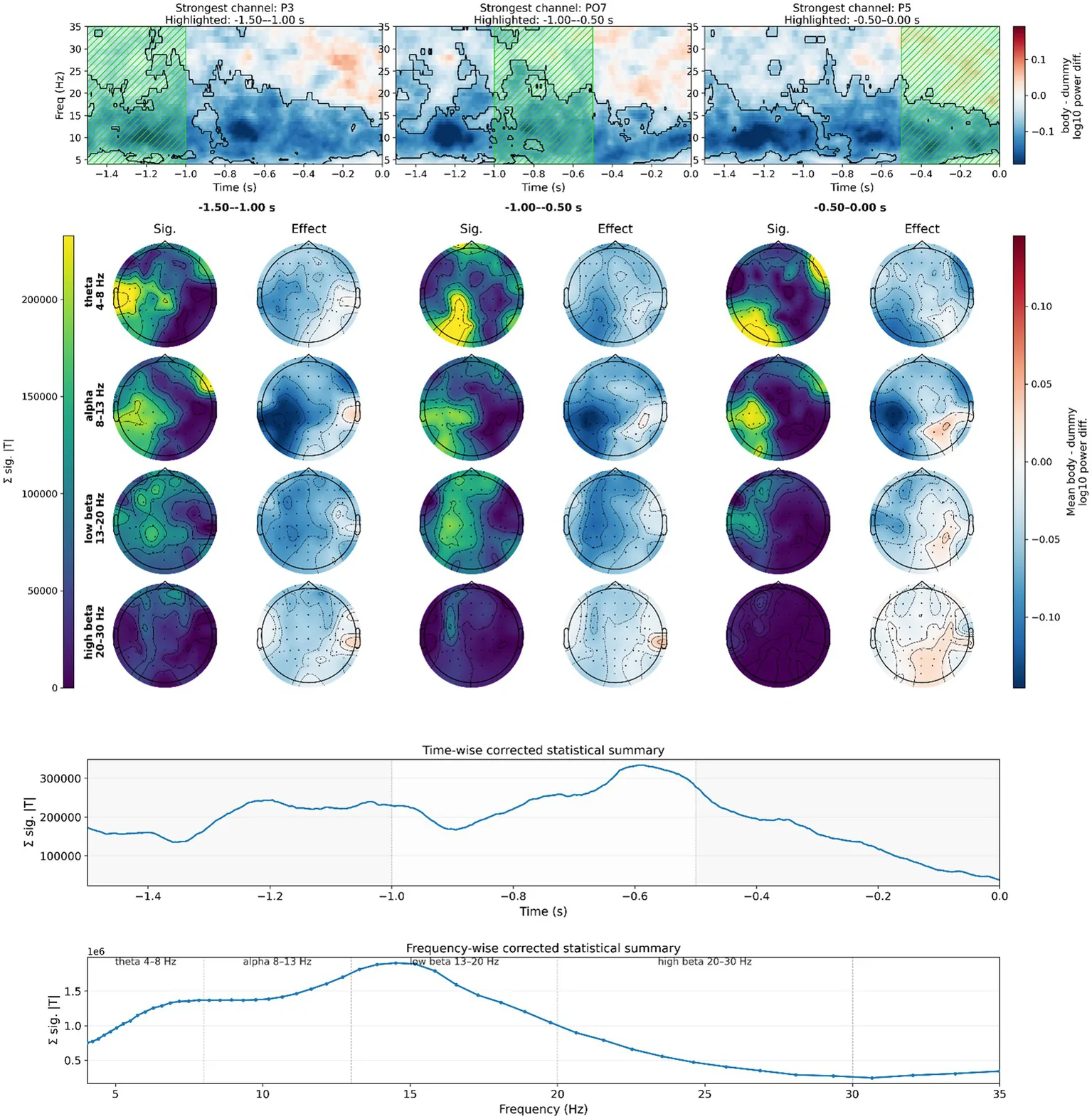
Whole-scalp TFCE-corrected time-frequency summary for the Body *vs*. Dummy contrast. Upper panels show the strongest-channel time-frequency maps across three response-locked windows. Green shading marks the highlighted time window, and contours indicate TFCE-corrected significant samples. Middle panels show band- and time-window-specific scalp maps of corrected statistical evidence (Sig.) and signed effect direction (Effect). The Sig. maps indicate where the corrected effect is statistically strongest, computed as the sum of significant absolute T-values within each band-window combination. The Effect maps show the signed group-level concept-minus-dummy power difference over the same band-window combination, indicating whether the direction of the effect is positive or negative. Lower panels show time-wise and frequency-wise corrected statistical summaries, computed as the sum of significant absolute T-values collapsed across channels and frequencies or channels and time, respectively.

**Figure 5:**
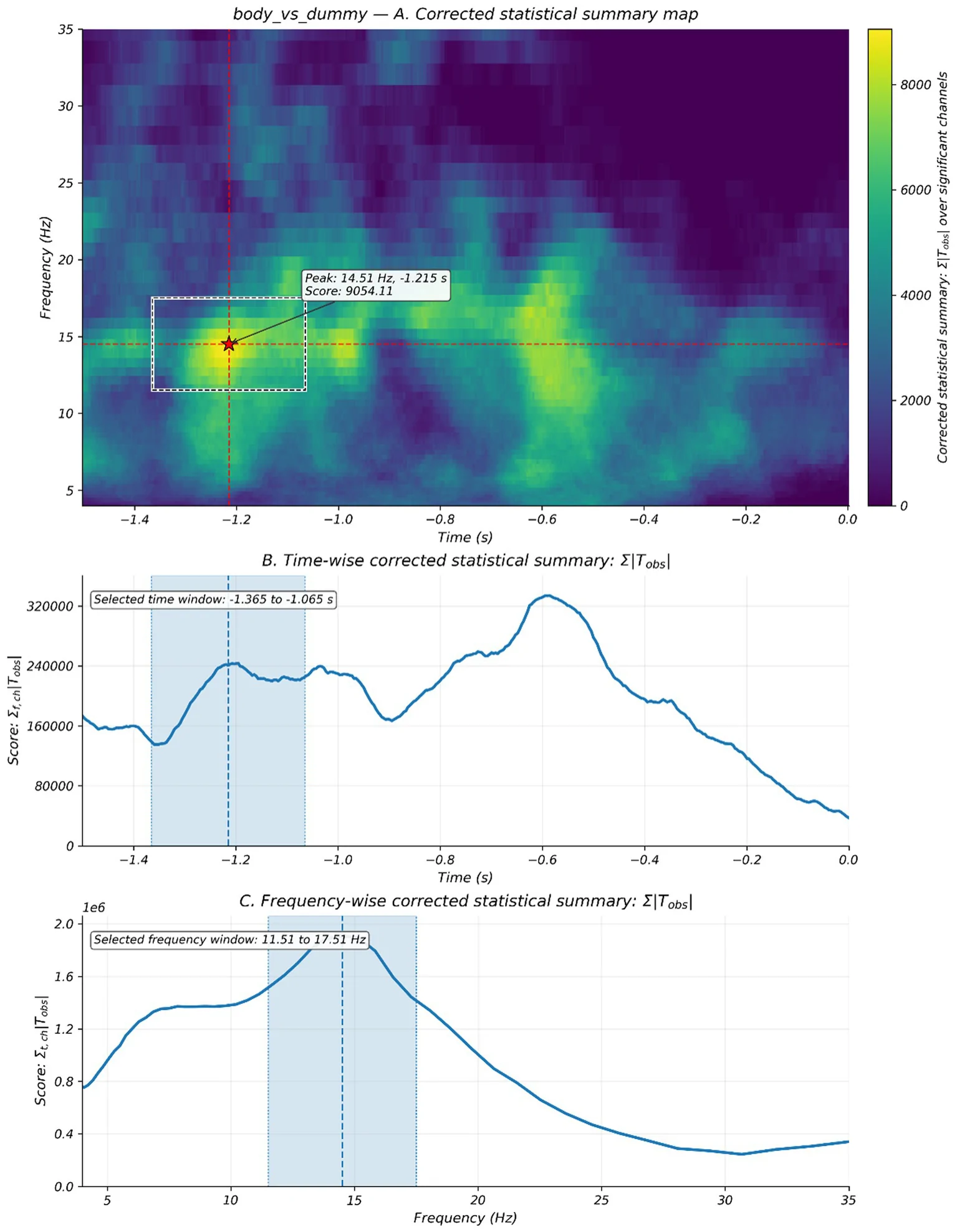
Peak-selection diagnostic for the Body *vs*. Dummy contrast. The frequency × time map shows corrected statistical evidence collapsed over channels, computed as the sum of significant absolute T-values. The selected peak occurred at approximately 14.51 Hz and −1.215 s, and the highlighted window was used for source-space projection.

**Figure 6:**
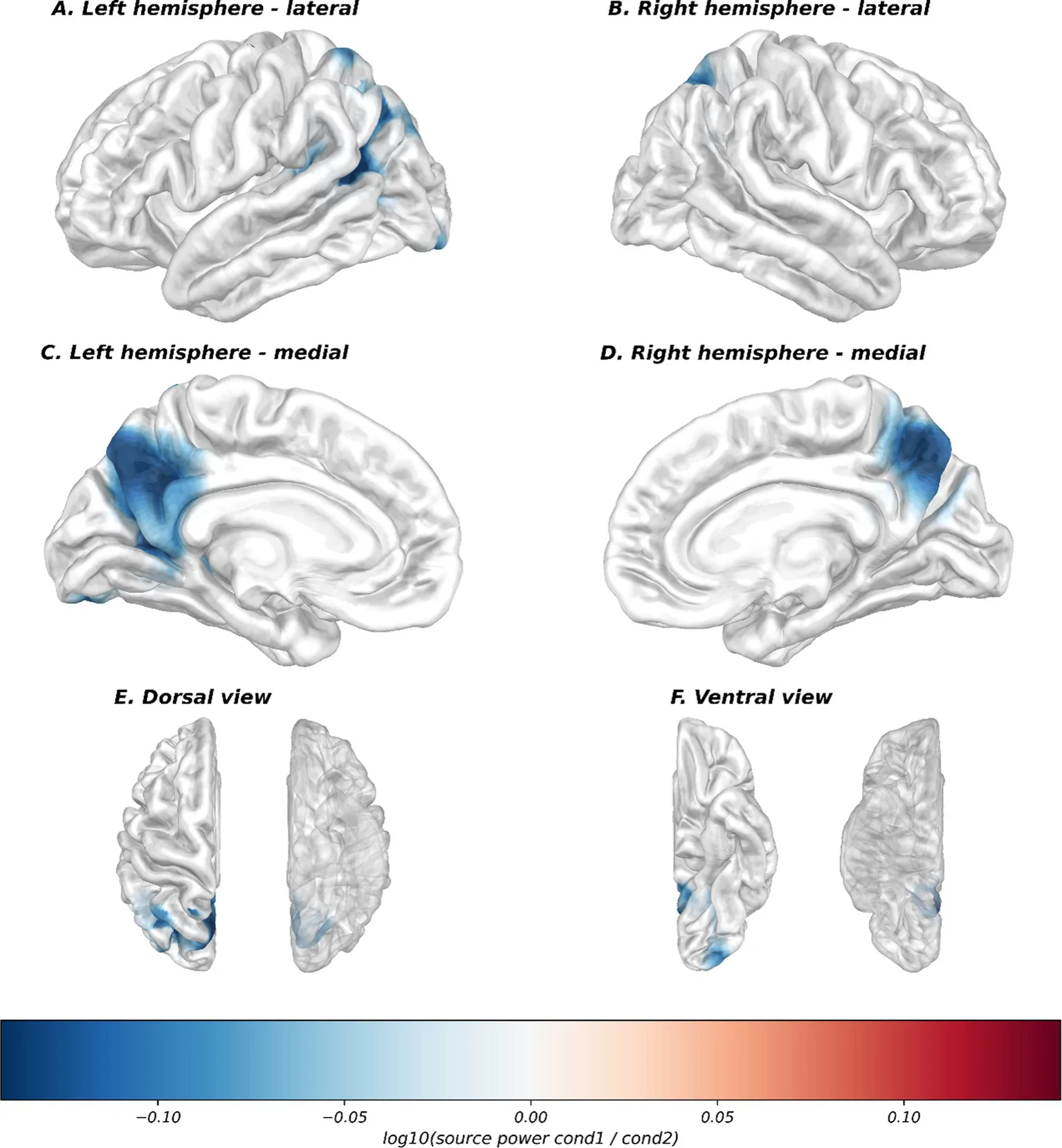
Exploratory LCMV source-space projection for the Body *vs*. Dummy contrast. The map shows the peak-defined low-beta concept-minus-dummy contrast. Blue values indicate lower source power for body concept trials relative to dummy trials.

**Figure 7:**
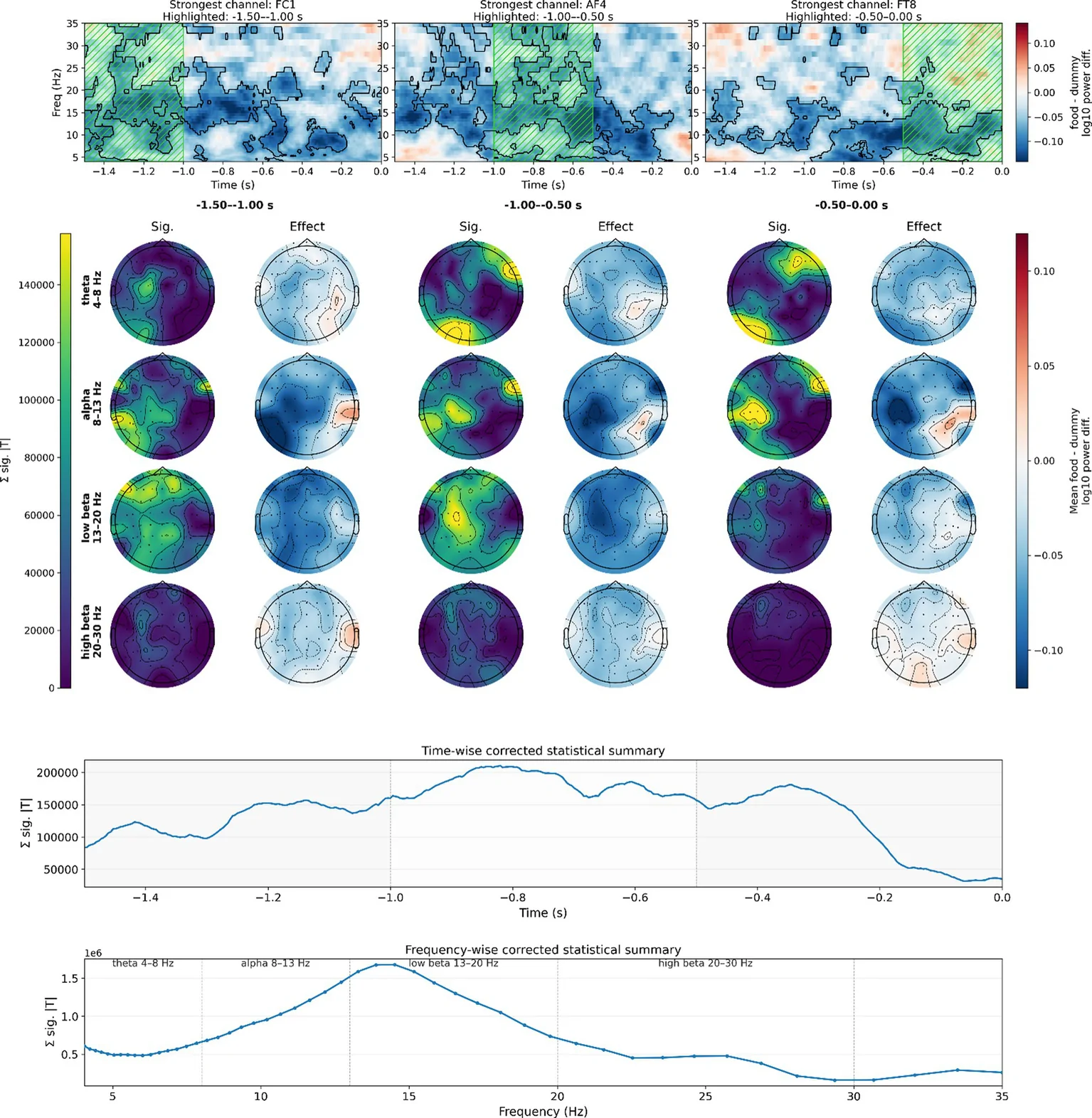
Whole-scalp TFCE-corrected time-frequency summary for the Food *vs*. Dummy contrast. The layout follows Figure 4. Corrected evidence was strongest in the low-beta range and was distributed across central, frontal, and posterior scalp regions. Signed effect maps indicate lower power for food concept trials relative to dummy trials.

**Figure 8:**
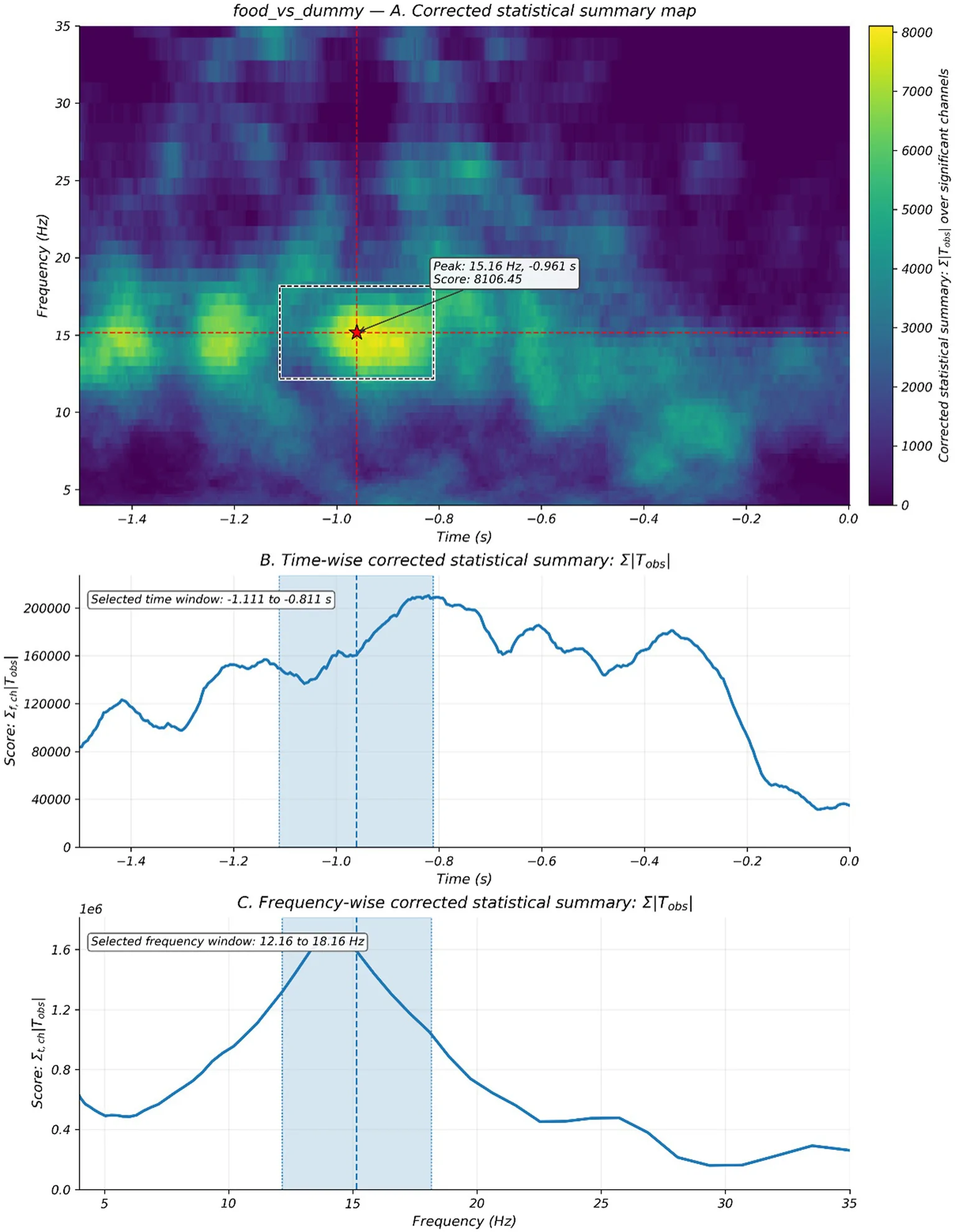
Peak-selection diagnostic for the Food *vs*. Dummy contrast. The corrected frequency × time score map was used to define the source-localisation window. The selected peak occurred at approximately 15.16 Hz and −0.961 s.

**Figure 9:**
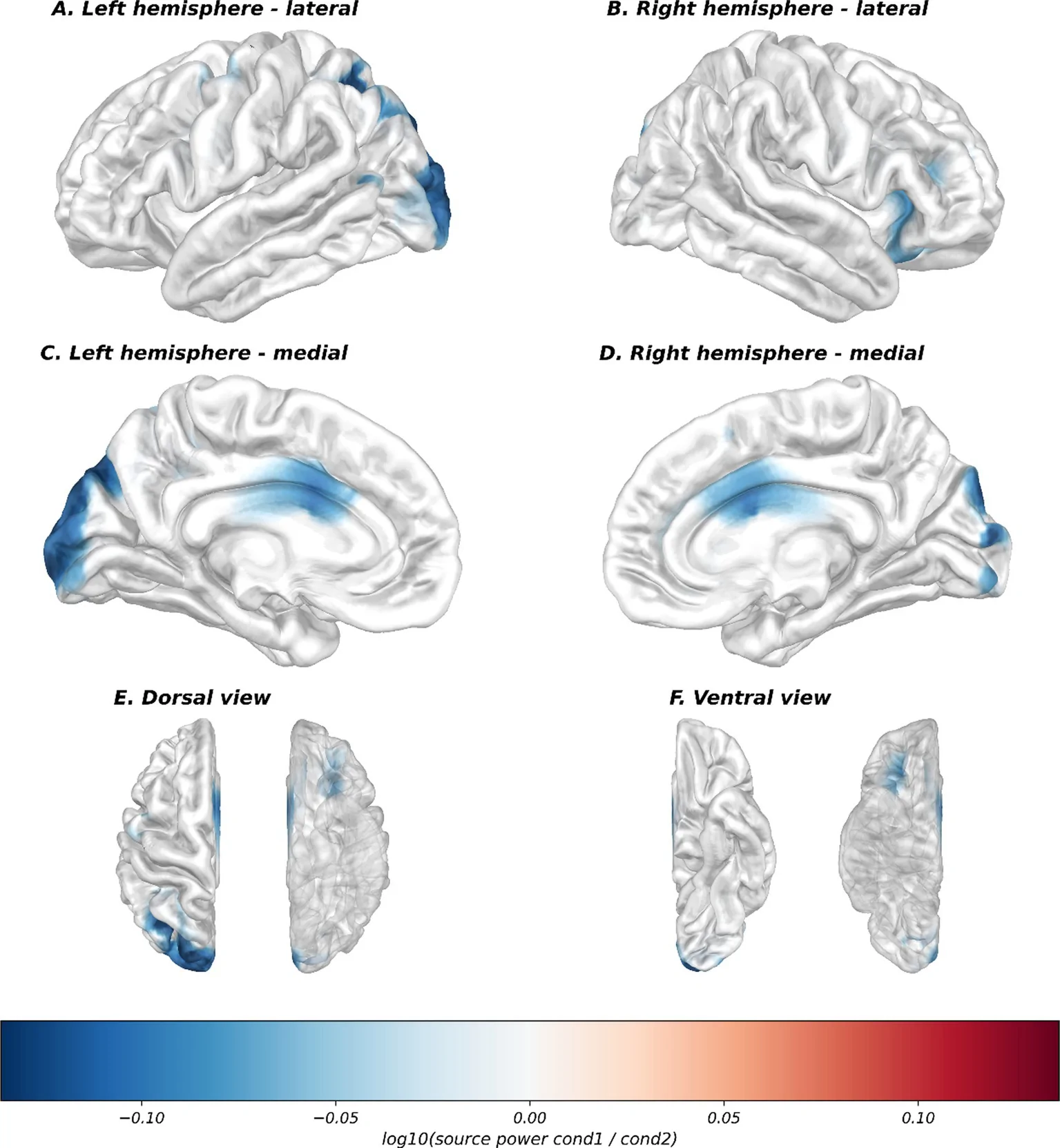
Exploratory LCMV source-space projection for the Food *vs*. Dummy contrast. The map shows the peak-defined low-beta food-minus-dummy contrast. Blue values indicate lower source power for food concept trials relative to dummy trials.

**Figure 10:**
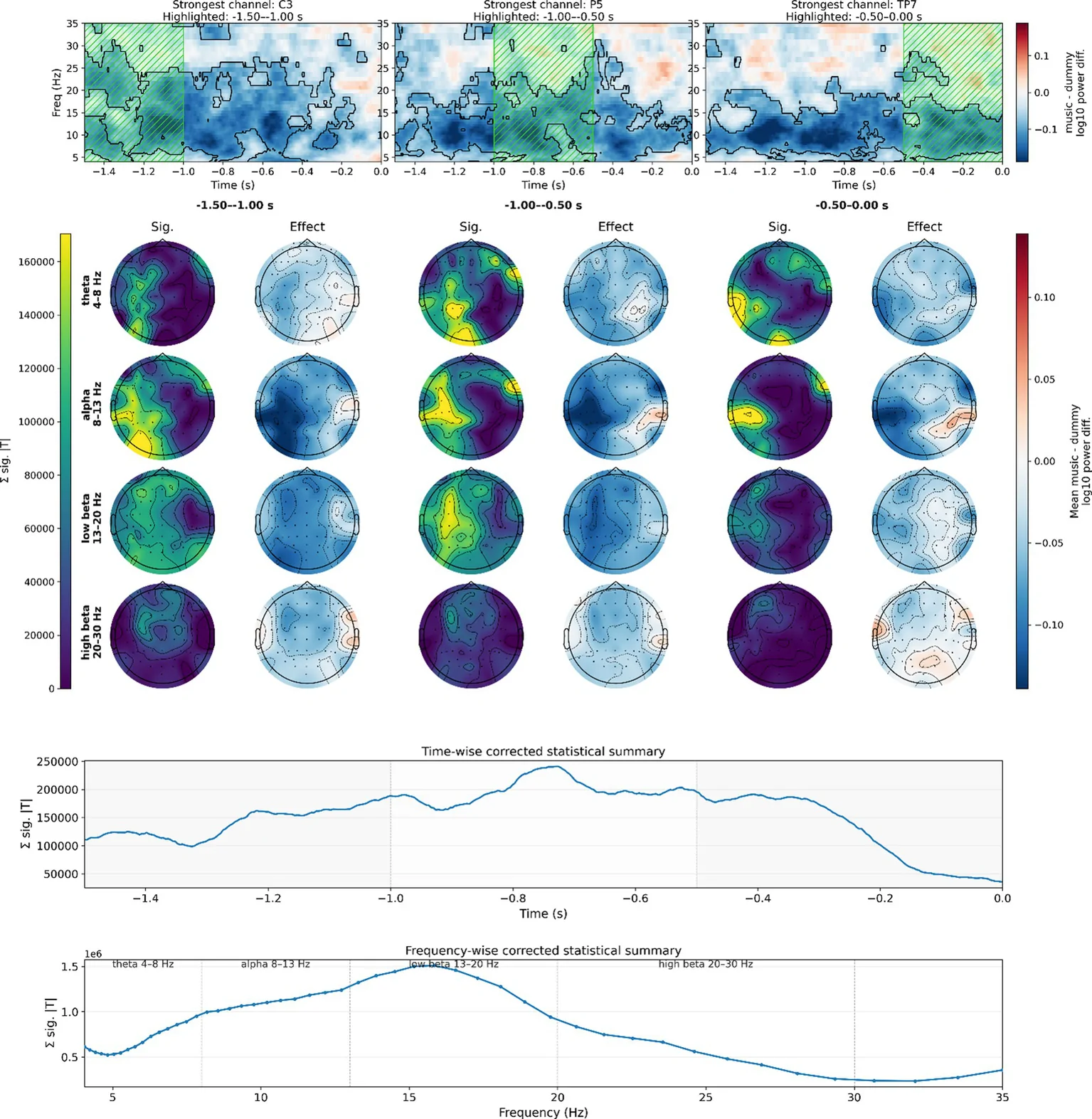
Whole-scalp TFCE-corrected time-frequency summary for the Music *vs*. Dummy contrast. The layout follows Figures 4, 7. The corrected effect peaked in the low-beta range and showed a left central/posterior emphasis. Signed effect maps indicate lower power for music concept trials relative to dummy trials.

**Figure 11:**
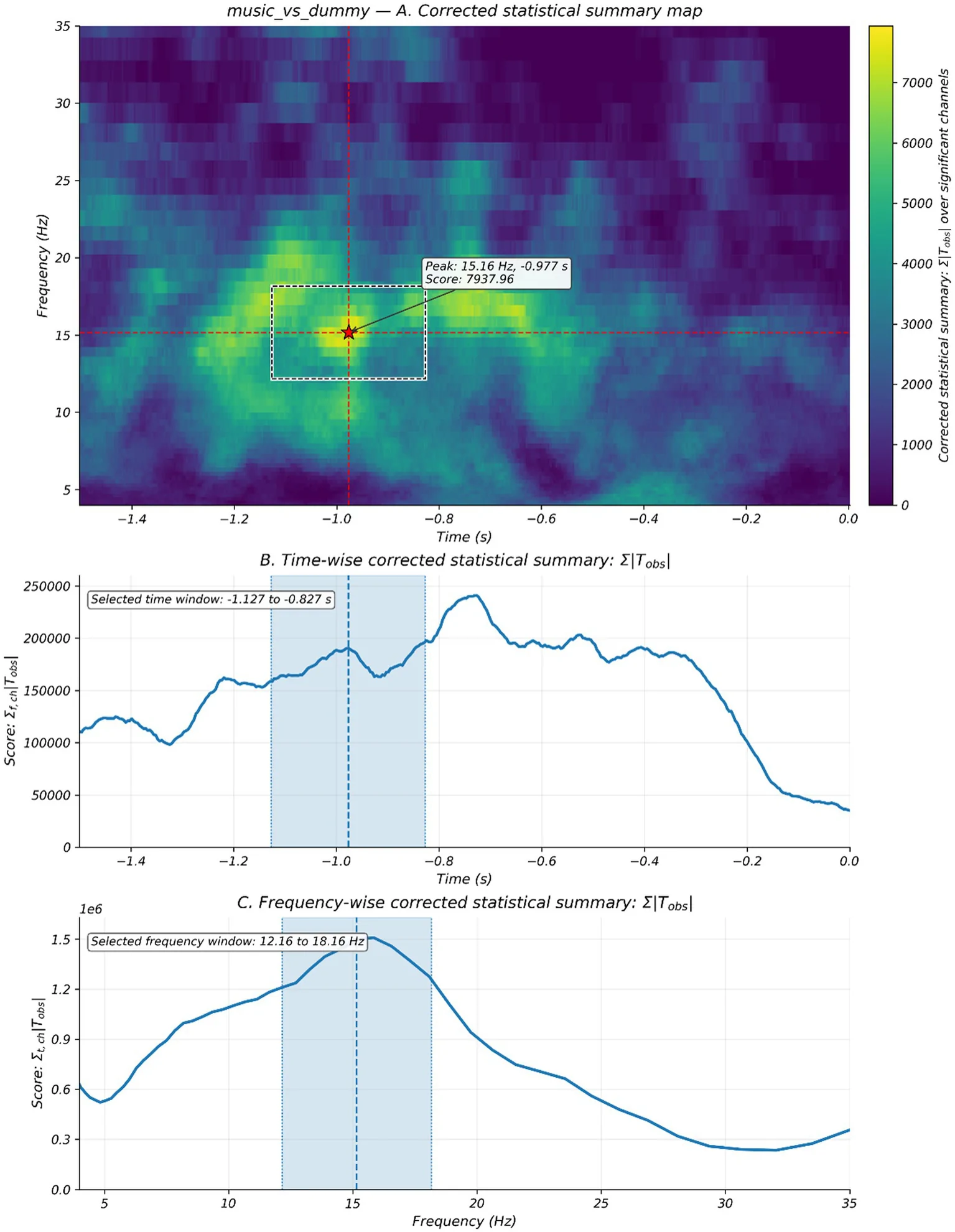
Peak-selection diagnostic for the Music *vs*. Dummy contrast. The corrected frequency × time score map was used to define the source-localisation window. The selected peak occurred at approximately 15.16 Hz and −0.977 s.

**Figure 12:**
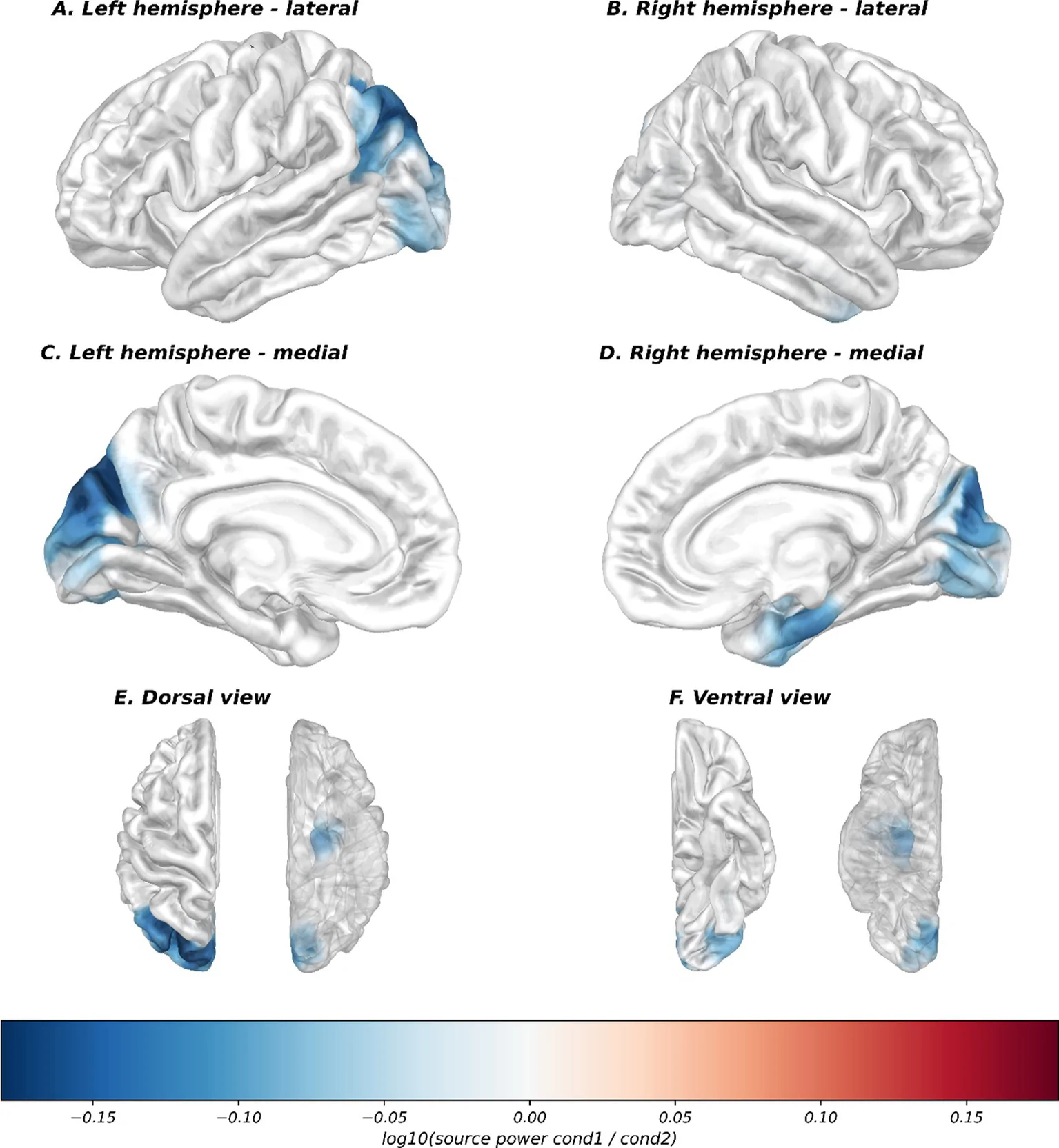
Exploratory LCMV source-space projection for the Music *vs*. Dummy contrast. The map shows the peak-defined low-beta music-minus-dummy contrast. Blue values indicate lower source power for music concept trials relative to dummy trials.

#### 3.2.1 Body *vs*. dummy

The Body *vs*. Dummy contrast produced the largest corrected effect among the three semantic categories, with 18.38% of tested channel × frequency × time samples surviving TFCE correction. The corrected statistical summary peaked at −0.586 s and 14.51 Hz, placing the dominant effect in the low-beta range during the pre-response interval. The strongest channels within the peak window were CP1, CP3, P1, PO7, P3, PO3, O1, C3, P7, and P5, indicating a predominantly centro-parietal and posterior scalp distribution.

Figure 4 shows the whole-scalp TFCE summary for this contrast. The same figure layout is used for the Food *vs*. Dummy and Music *vs*. Dummy summaries in Figures 7, 10. The upper row contains the strongest-channel time-frequency maps for three consecutive response-locked windows: −1.50 to −1.00 s, −1.00 to −0.50 s, and −0.50 to 0.00 s. For each window, the representative channel was selected as the channel with the largest corrected statistical evidence within that time interval, quantified as the sum of significant absolute T-values. The colour scale in these panels shows the signed concept-minus-dummy time-frequency power difference at that channel, while the contour outlines TFCE-corrected significant samples. The green shaded rectangle marks the time window represented by that panel. The middle section of the figure contains band-by-window scalp topographies. The rows correspond to predefined frequency bands: theta 4–8 Hz, alpha 8–13 Hz, low beta 13–20 Hz, and high beta 20–30 Hz. For each band and time window, the Sig. map summarises corrected statistical evidence at each electrode, computed as the sum of significant absolute T-values within that band-window combination.

The lower panels provide one-dimensional summaries of the corrected statistical evidence. The time-wise summary collapses significant absolute T-values across channels and frequencies, showing when corrected evidence is strongest. The frequency-wise summary collapses significant absolute T-values across channels and time, showing which frequencies contribute most strongly to the corrected effect. These lower summaries are used to identify the table-level peak time and peak frequency (Table 1). In the Body *vs*. Dummy contrast, the whole-scalp summary showed broad corrected evidence across the pre-response interval, with the strongest evidence in the low-beta range and with prominent centro-parietal/posterior scalp involvement. The signed effect maps were predominantly negative, consistent with the Body < Dummy direction reported in Table 1.

For source-space projection, a separate peak-selection diagnostic was used to define a compact time-frequency window (Figure 5). The diagnostic plot collapses corrected statistical evidence over channels to form a frequency × time score map, where each point reflects the sum of significant absolute T-values across channels. The peak selected for source localisation is the maximum of this corrected frequency × time score map. A local time-frequency window centred on this peak is then used for LCMV source-space projection. This procedure links the source maps to the strongest corrected sensor-level time-frequency evidence rather than to an arbitrary frequency band or latency interval. The same peak-selection logic is used for Figures 8, 11. For Body *vs*. Dummy, the diagnostic peak occurred at approximately 14.51 Hz and −1.215 s.

The corresponding exploratory LCMV source-space projection is shown in Figure 6. The source map represents the peak-defined low-beta Body *vs*. Dummy contrast and is displayed as an un-thresholded descriptive projection on *fsaverage* pial anatomy. Blue values indicate lower source power for body concept trials relative to dummy trials, consistent with the negative sensor-level TFCE direction. The strongest projected effects were visible over the posterior and medial cortical surfaces, with additional lateralised posterior contributions. Because these maps are derived from scalp EEG, they should be interpreted as approximate source-space summaries of the sensor-level effect rather than precise anatomical localisations. This interpretive constraint also applies to the source projections in Figures 9, 12.

#### 3.2.2 Food *vs*. dummy

The Food *vs*. Dummy contrast produced a TFCE-corrected effect occupying 12.28% of the tested channel × frequency × time space. The corrected statistical summary peaked at −0.852 s and 14.41 Hz, again indicating a low-beta effect during the pre-response interval. The strongest channels within the peak window were CP1, C3, CP3, AF4, O1, AF8, F4, P1, FT8, and C1. Compared with the body contrast, the food contrast showed a broader spatial distribution involving central, frontal, and posterior sites.

Using the same whole-scalp TFCE summary layout introduced in Figure 4, Figure 7 shows that the Food *vs*. Dummy effect was distributed across central, frontal, and posterior scalp regions. The low-beta band again provided the strongest frequency-wise contribution, and the signed effect maps were predominantly negative, consistent with the Food < Dummy direction reported in Table 1.

The peak-selection diagnostic identified a low-beta peak at approximately 15.16 Hz and −0.961 s (Figure 8).

The source-space projection for this peak-defined Food *vs*. Dummy window showed predominantly negative concept-minus-dummy contrast values. The projected pattern was distributed across posterior, lateral, and medial cortical surfaces, consistent with the broad sensor-level topography observed in Figure 7.

#### 3.2.3 Music *vs*. dummy

The Music *vs*. Dummy contrast showed a TFCE-corrected effect occupying 14.99% of the tested channel × frequency × time space. The corrected statistical summary peaked at −0.762 s and 15.84 Hz, again within the low-beta range. The strongest channels within the peak window were C3, P3, CP3, P5, P1, FC3, CP5, PO7, P7, and PO3, indicating a left central and posterior emphasis.

Using the same whole-scalp summary format as Figures 4 and 7, Figure 10 shows that the Music *vs*. Dummy effect shared the low-beta profile observed in the other contrasts, but with a more left centro-parietal/posterior scalp distribution. The signed effect maps again showed a negative concept-minus-dummy direction, consistent with Music < Dummy in Table 1.

The peak-selection diagnostic identified a low-beta peak at approximately 15.16 Hz and −0.977 s (Figure 11).

The corresponding source-space projection showed predominantly negative concept-minus-dummy source-power contrast values (Figure 12), with visible posterior, lateral, medial, and ventral contributions. This projection was broadly consistent with the left central/posterior sensor-level pattern observed in Figure 10, while remaining exploratory and descriptive rather than anatomically definitive.

### 3.3 Response-latency and motor-preparation control analyses

To assess whether the concept *vs*. dummy low-beta effect could be explained by response timing or differential motor preparation, additional control analyses were conducted across all retained epochs with valid response-latency information (*n* = 3,408). Response latency was defined as the interval between trial-audio onset and the spacebar press and was related to mean log10-transformed low-beta power in the 13–20 Hz band within the same −1.5 to 0 s response-locked interval used in the principal analysis.

Response latency showed no meaningful relationship with low-beta power when averaged across either the complete 64-channel montage (Pearson’s r = 0.009, *p* = 0.61) or the 27 left-hemisphere channels (*r* = −0.003, *p* = 0.85). As shown in Figure 13, the pooled regression slopes were effectively flat in both analyses, despite the clear difference in the response-latency distributions of concept and dummy trials. Spearman correlations led to the same substantive conclusion. These pooled epoch-level analyses were intended as descriptive control analyses of whether response latency accounted for a meaningful proportion of low-beta variation.

**Figure 13:**
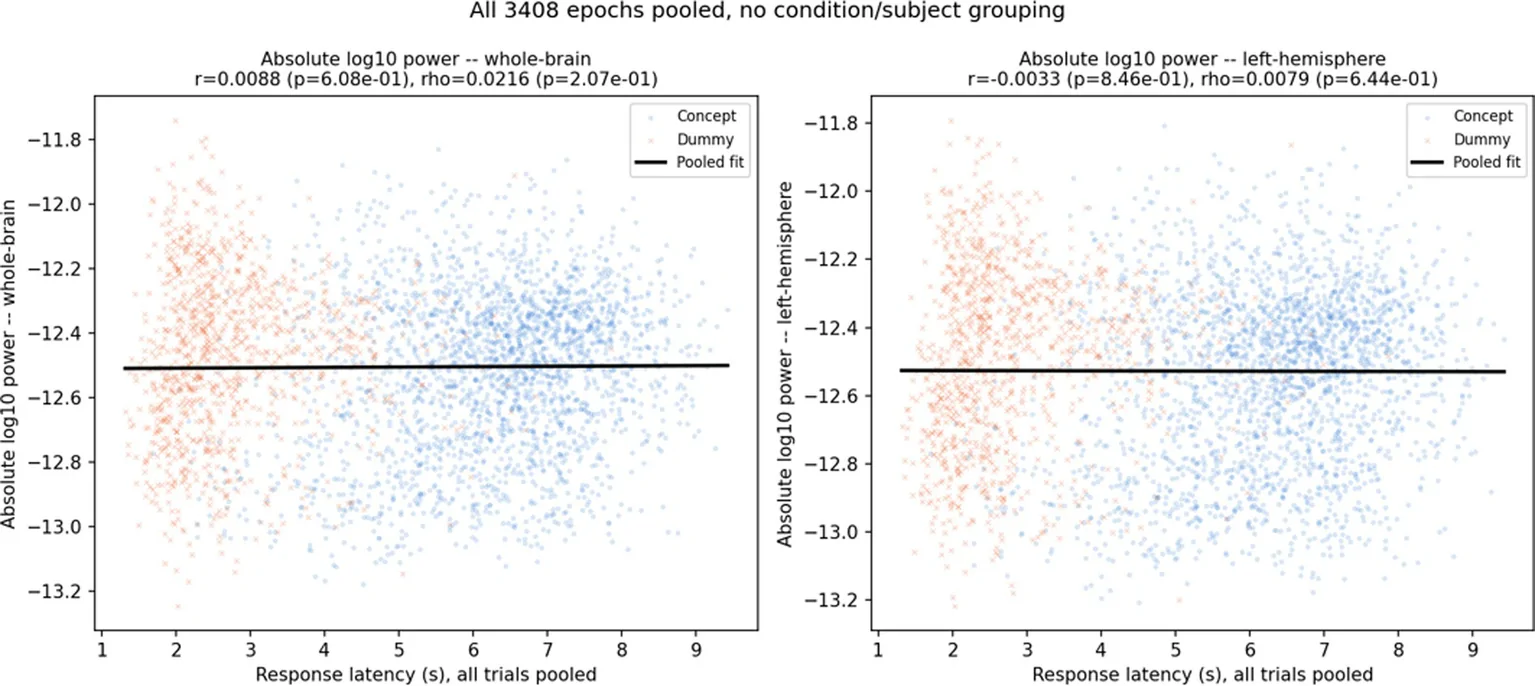
Relationship between response latency and low-beta power. Pooled associations between response latency and mean log10-transformed low-beta power within the 13–20 Hz band and the −1.5 to 0 s response-locked analysis window. Power was averaged across the complete 64-channel montage in the left panel and across the 27 left-hemisphere channels in the right panel. Each point represents one retained epoch (*n* = 3,408), with concept and dummy trials displayed separately for visualisation. The black line represents the regression fit across all retained epochs. Response latency showed no meaningful association with low-beta power at either the whole-scalp level (Pearson’s *r* = 0.009, *p* = 0.61) or the left-hemisphere level (*r* = −0.003, *p* = 0.85). The pooled epoch-level correlations are presented as descriptive control analyses rather than participant-level inferential estimates.

To determine whether spatially localised associations were obscured by channel averaging, the analysis was repeated independently at each of the 64 electrodes (Supplementary Figure S1). Ten electrodes survived Bonferroni correction across the 64 tests: Iz, F8, F4, P9, P6, P4, PO4, P8, P2, and PO8. However, the corresponding associations were small, with response latency explaining only 0.35–0.74% of the variance in low-beta power. The correlations also differed in direction across channels, with negative associations at Iz, F8, F4, and P9 and positive associations at P6, P4, PO4, P8, P2, and PO8. None of the surviving electrodes fell within the predefined central sensorimotor set, and only F4 overlapped with the highest-ranked electrodes contributing to the principal concept *vs.* dummy effects.

Motor preparation was examined separately by comparing pre-response 8–13 Hz mu activity between concept and dummy trials at C3, C4, and Cz (Supplementary Figure S2). Both conditions showed comparable, overlapping mu desynchronisation approaching the spacebar response, with no cluster-corrected condition difference at any of the three channels. Additional participant-level and response-latency-tertile analyses likewise provided no consistent evidence that motor preparation differed systematically between conditions or varied as a function of response latency.

Together, these results indicate that the principal low-beta contrast was not meaningfully predicted by response latency and was not accompanied by a detectable difference in conventional sensorimotor preparation. Trial-by-trial confidence ratings were not independently collected, however, and the respective contributions of confidence development and other decision-related processes within the broader semantic-retrieval cascade could not be estimated separately.

### 3.4 Participant-level directional consistency

Participant-level consistency was assessed by extracting each participant’s mean signed effect from the TFCE-corrected significant mask. For all three contrasts, every participant showed the same negative direction as the group-level effect. Directional consistency was therefore 13/13 participants for Body *vs*. Dummy, Food *vs*. Dummy, and Music *vs*. Dummy, with one-sided binomial directional-consistency *p* < 0.001 in each case. Figure 14 visualises these extracted participant-level effects across the three contrasts, with all participant-level values below zero confirming that the negative concept-minus-dummy modulation was consistent across participants and semantic categories.

**Figure 14:**
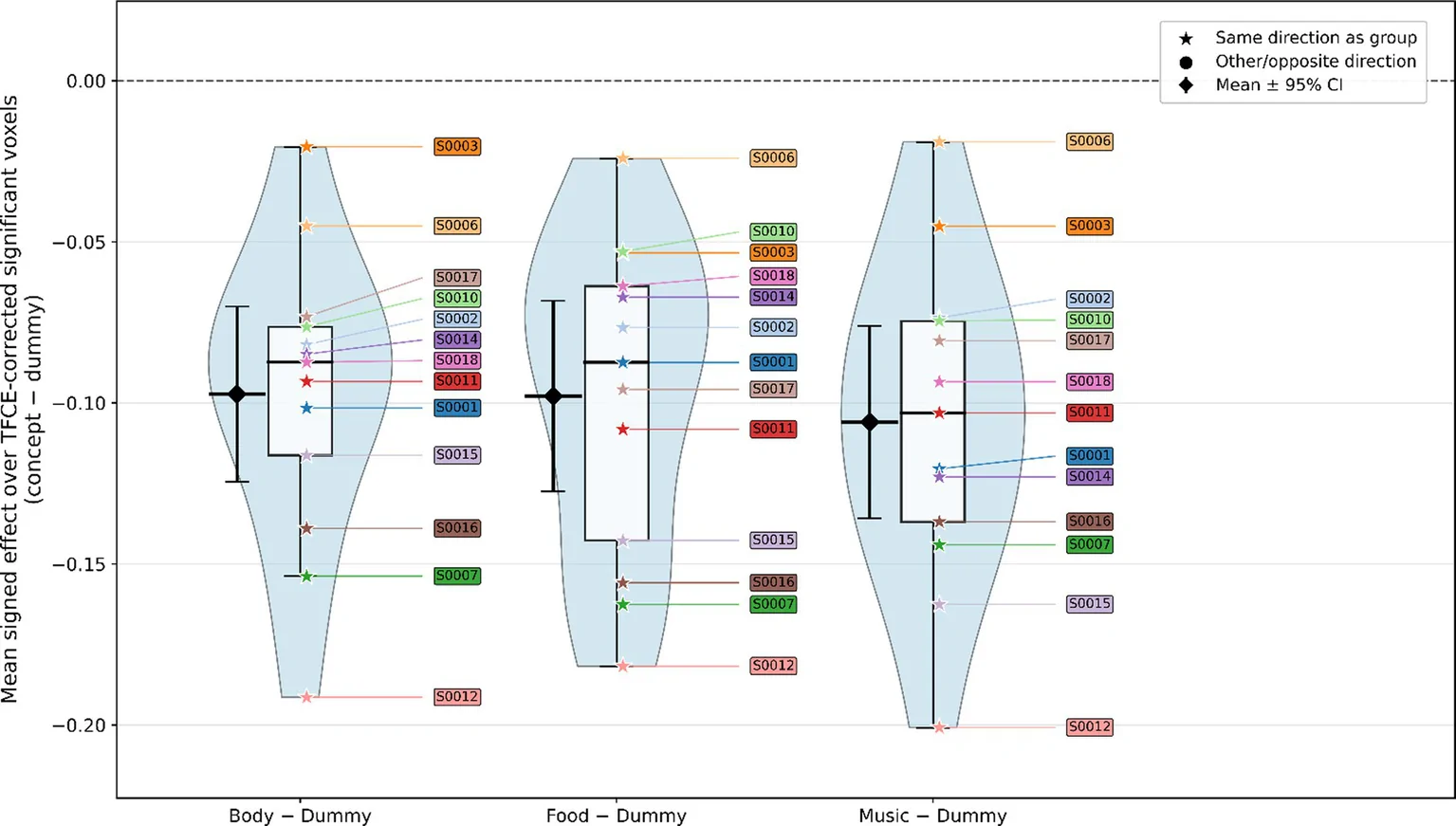
Participant-level consistency of TFCE-corrected concept *vs*. dummy effects across the three semantic categories. Each point represents one participant’s mean signed effect extracted from the TFCE-corrected significant mask for that contrast. Stars indicate participants whose effect direction matched the group-level direction, and diamonds show the group mean with 95% confidence intervals. All participant-level values were below zero across all three contrasts, confirming complete directional consistency of the negative concept-minus-dummy modulation.

## 4 Discussion

In this study we introduced GRASP as a response-locked auditory semantic retrieval paradigm designed to characterise the spatio-spectro-temporal properties of meaningful concept processing in scalp EEG. Across all three planned concept *vs.* dummy contrasts, Body *vs*. Dummy, Food *vs*. Dummy, and Music *vs*. Dummy, the dominant effect was a robust reduction of low-beta power for concept trials relative to pseudo-word dummy trials. This effect was observed before the behavioural response, peaked consistently between approximately 14 and 16 Hz, and showed complete participant-level directional consistency, with all 13 participants exhibiting the same negative concept-minus-dummy direction in each contrast. The spatial distribution differed across semantic categories, but the frequency profile and direction of the effect were strikingly stable. This pattern suggests that the concept *vs.* dummy contrast captures a cross-participant-consistent electrophysiological signature of meaningful semantic retrieval, rather than an idiosyncratic or category-specific effect driven by a subset of individuals.

### 4.1 Validation of the pseudo-word reference condition

Our central aim with GRASP was to provide a principled low-semantic reference condition against which concept-related EEG activity could be characterised. The present findings support this aim. The dummy trials were not a passive rest condition, rather, they preserved auditory stimulation, sentence-like structure, task engagement, button pressing, and response typing requirements, while substantially reducing recoverable lexical-semantic content. The observation of robust TFCE-corrected concept *vs.* dummy effects therefore supports the use of phonologically matched pseudo-word sentences as an active low-semantic baseline for isolating meaningful retrieval processes.

This interpretation is consistent with evidence from jabberwocky sentence paradigms (Desbordes et al., 2023). Hahne and Jescheniak (2001) showed that auditory sentences in which content words were replaced by pseudo-words can preserve syntactic processing while reducing lexical-semantic integration, with regular content words eliciting an N400 response that pseudo-word placeholders did not elicit in syntactically correct sentences (Hahne and Jescheniak, 2001). More broadly, the N400 literature establishes that electrophysiological responses are highly sensitive to the availability, expectancy, and integration of meaning across spoken and written language (Kutas and Federmeier, 2011). The present response-locked time-frequency effects reported here should not be described as a direct N400 replication, because the analysis was aligned to the participant’s spacebar response rather than to individual word onsets. However, our results are compatible with the same general principle, namely, that when lexical-semantic content is recoverable and progressively integrated, electrophysiological activity differs reliably from pseudo-word input that preserves surface linguistic structure but lacks meaningful content.

The findings also extend recent work showing that meaningful and jabberwocky sentences differ in their representational geometry. Desbordes et al. (2023) reported that meaningful sentences elicit higher representational dimensionality than jabberwocky sentences in MEG and intracranial EEG, and that normal *vs*. jabberwocky sentences can be separated through dynamic patterns involving temporal/parietal, frontal, and sentence-final signals. GRASP differs from that study in several important respects, notably, it used auditory definitions rather than visually presented word sequences, scalp EEG rather than MEG/sEEG, healthy participants rather than a clinical intracranial sample, and response-locked rather than word-locked analysis. Nevertheless, our results provide convergent evidence that the meaningful *vs.* pseudo-word distinction is recoverable from electrophysiological data. The key additional contribution is translational, here GRASP demonstrates that this distinction can be detected non-invasively, during active semantic retrieval, and with complete directional consistency across participants.

### 4.2 Low-beta desynchronisation as a marker of semantic retrieval

The most important spectral finding was the dominance of low-beta activity. The corrected effects peaked at 14.51 Hz for Body *vs*. Dummy, 14.41 Hz for Food *vs*. Dummy, and 15.84 Hz for Music *vs*. Dummy, with concept trials consistently showing lower power than dummy trials. This pattern is best understood as a low-beta desynchronisation associated with meaningful concept retrieval.

This interpretation is strongly compatible with broader EEG and MEG literature. Event-related desynchronisation has long been interpreted as a marker of task-related cortical engagement, particularly when reduced rhythmic power reflects the release of a cortical network from an idling or synchronised state into active information processing (Pfurtscheller and Lopes da Silva, 1999). In memory research, alpha/beta power decreases have been linked to richer item-specific encoding and retrieval, leading to the “information via desynchronisation” hypothesis. According to this account, decreases in oscillatory power can support the representation of more detailed information by reducing synchrony and allowing more flexible neural coding (Hanslmayr et al., 2012). This framework maps well onto the present contrast as here concept trials required participants to recover a specific meaningful target from accumulating auditory clues, whereas dummy trials required recognition that no meaningful target was recoverable. The stronger low-beta reduction for concept trials may therefore index the richer representational state associated with meaningful retrieval.

Further, this finding is also consistent with language-specific accounts of beta oscillations. Lewis and colleagues proposed that beta activity during sentence-level comprehension is linked to the maintenance or change of the neural network configuration used to construct and represent sentence meaning, as well as to top-down predictions about upcoming linguistic input (Lewis et al., 2016). From this perspective, the reduction of low-beta power in concept trials may reflect the updating or reconfiguration of semantic networks as participants move from partial clue information toward a recoverable concept. In contrast, dummy trials may maintain a more stable monitoring state because the pseudo-word stream does not support the same progressive convergence onto a coherent semantic representation.

The desynchronisation may also reflect an active semantic search state that is sustained while evidence accumulates and collapses once the target concept is identified. This interpretation is consistent with the dimensionality account of Desbordes et al. (2023), who showed that meaningful sentences elicit substantially higher representational dimensionality than pseudo-word sentences, with the dimensional signature evolving dynamically across the pre-response window. The progressive low-beta desynchronisation observed here may reflect the same process, an expanding representational state that collapses at the moment of concept identification.

The response-locked timing of the effect required consideration of whether systematic differences in response latency or motor preparation could account for the low-beta contrast. The additional control analyses did not support either explanation. Across 3,408 retained epochs, response latency was unrelated to low-beta power when averaged across the complete scalp montage or the left hemisphere. Although 10 individual channels showed Bonferroni-corrected latency–power associations, each explained less than 1% of the variance, the directions of association differed across electrodes, and the surviving channels were not concentrated over the conventional sensorimotor region and showed only minimal overlap with the highest-ranked electrodes contributing to the principal concept *vs.* dummy effects. Moreover, concept and dummy trials showed overlapping pre-response mu desynchronisation at C3, C4, and Cz, with no cluster-corrected condition difference.

These findings make a simple response-timing or conventional motor-preparation account of the low-beta effect unlikely. They do not, however, allow the respective contributions of confidence development, evidence accumulation, and decision formation to be fully separated. Trial-by-trial confidence was not independently measured, and concept and dummy trials necessarily involved different decision trajectories. The low-beta modulation is therefore most appropriately interpreted as reflecting neural processing associated with meaningful semantic retrieval, understood as a distributed higher-order cognitive operation rather than a unitary process. Successful concept retrieval necessarily recruits multiple interacting and regionally distributed processes, including linguistic integration, evidence accumulation, lexical access, target selection, and decision formation. The present design cannot fully separate the respective contributions of these component processes, but their involvement should not be regarded as independent of semantic retrieval.

Our findings also suggest a limited parallel with the motor-imagery literature that may inform future research. In motor-imagery BCIs, imagined movement is commonly accompanied by event-related desynchronisation of mu and beta rhythms over sensorimotor regions. The spatial distribution and lateralisation of these ERD/ERS patterns can contribute to the discrimination of commonly used motor-imagery classes, including left-hand, right-hand, foot, and tongue imagery (Pfurtscheller et al., 2006). The present findings suggest a partial analogy in the semantic domain, whereby concept retrieval is accompanied by a task-related reduction in low-beta power, consistent with low-beta desynchronisation. However, this analogy concerns the broader principle of ideation-related oscillatory desynchronisation rather than a direct correspondence between motor somatotopy and semantic topography. The parallel is necessarily limited in two important respects. First, the contrast we define is concept *vs*. dummy rather than concept *vs*. concept. Our previous work demonstrated above-chance cross-participant concept *vs*. concept decoding using the same three semantic categories, achieving mean accuracies of 73.4, 70.7, and 68.2% for Body *vs*. Food, Body *vs*. Music, and Food *vs*. Music, respectively (Podmore et al., 2026). Critically, those categories were selected precisely because they represent the most separable semantic domains in both word2vec embedding space and fMRI activation space (Radman et al., 2025), meaning that even under maximally favourable conditions for concept *vs*. concept discrimination, performance remained modest. The present contrast therefore targets a more tractable and participant-invariant signal by focusing on the high-semantic *vs*. low-semantic boundary rather than graded representational differences between categories.

Second, while the somatotopic organisation of sensorimotor cortex provides a well-understood neural substrate for motor imagery ERD, the equivalent semantic topography is considerably less resolved. Recent work has begun to characterise distributed semantic maps across the cortex (Huth et al., 2016), but the degree of spatial specificity available for semantic decoding remains substantially lower than that available for motor imagery. Concept-level differentiation in our data emerges principally along the spatial axis, with the frequency and temporal profiles remaining strikingly consistent across categories while the channel-wise topographic patterns diverge in ways that reflect the modal grounding of each concept. Improving the spatial resolution of this approach will therefore require higher-density electrode arrays to better resolve these distributed topographic differences.

### 4.3 Spatial distribution and the hub-and-spoke framework

Our first hypothesis predicted a bilateral anterior temporal hub maximum common to all three concept *vs*. dummy contrasts, reflecting systematic hub engagement during meaningful semantic retrieval. Our present findings do not support this prediction. Neither the sensor-level topographies nor the exploratory LCMV source projections showed a focal anterior temporal maximum in any of the three category-specific contrasts. Instead, sensor-level effects were distributed across left-lateralised centro-parietal, posterior, and frontal sites, with category-sensitive topographic differences. The exploratory source projections were consistent with this pattern, showing predominantly posterior and medial parieto-occipital distributions across all three contrasts, with food showing the most bilateral posterior involvement and body and music showing stronger left-lateralised posterior contributions.

The overall left-weighted, but category-dependent, scalp distribution requires consideration independently of the absence of an anterior temporal maximum. The bilateral organisation proposed by the hub-and-spoke framework concerns the underlying neural architecture supporting conceptual representations and does not necessarily predict that every electrophysiological contrast involving semantic processing will produce a spatially symmetric scalp pattern (Lambon Ralph et al., 2017). The present contrast did not isolate amodal conceptual representation alone. Rather, participants listened to spoken definitions, integrated successive linguistic clues, accumulated evidence, accessed lexical and conceptual information, selected among candidate concepts, and prepared a response once a target became sufficiently recoverable. These processes are integral components of the broader semantic-retrieval cascade and may preferentially recruit left-weighted language and semantic-control networks.

This interpretation is broadly compatible with the distribution of the principal effects. The strongest sensor-level contributions were located predominantly over left centro-parietal and posterior regions for the Body and Music contrasts, whereas the Food contrast showed a broader distribution with additional frontal involvement. The observed pattern may therefore reflect the interaction of distributed conceptual representations with left-weighted linguistic, integrative, and controlled-selection processes involved in combining spoken clues and converging on a lexical target. Accordingly, the observed asymmetry should not be interpreted as evidence that semantic representations themselves are exclusively left-lateralised or that the bilateral hub-and-spoke architecture is invalid. Rather, it suggests that the component of meaningful semantic retrieval most readily detectable using scalp EEG in the present auditory paradigm may primarily reflect the left-weighted language-mediated processes through which the broader semantic system is accessed.

The absence of a focal anterior temporal maximum should likewise not be interpreted as evidence against the hub-and-spoke framework, but it requires careful methodological consideration. The dummy condition is a low-semantic state, not a zero-semantic state. Pseudo-word sentences preserve sentence-like auditory structure, function words, prosody, and task engagement, meaning that some degree of semantic processing is plausibly occurring in both conditions. The ATL hub is therefore likely engaged during both concept and dummy trials. Previous studies that successfully identified ATL hub involvement used genuinely zero-semantic or non-linguistic baselines, lesion and disruption methods including repetitive transcranial magnetic stimulation (TMS) in healthy participants (Lambon Ralph et al., 2017), and the pan-modal semantic degradation observed in semantic dementia (Patterson et al., 2007).

These approaches maximise the signal-to-noise ratio for hub detection by creating a large differential between conditions or by directly disrupting hub function. The present contrast, which subtracts a low-semantic condition from a high-semantic one using scalp EEG without individual MRIs, is unlikely to resolve a differential hub contribution even if one exists. The absence of an anterior temporal maximum therefore reflects both the reduced dynamic range of the concept *vs*. dummy contrast relative to the hub signal and the known insensitivity of scalp EEG to deep ventral anterior temporal generators. The observed contrast may therefore be weighted toward differential engagement of distributed linguistic, associative, and modality-sensitive spoke regions during meaningful concept retrieval relative to pseudo-word input, broadly consistent with the hub-and-spoke model (Patterson et al., 2007; Lambon Ralph et al., 2017). The large-scale cortical mapping evidence of Huth et al. (2016), which shows semantic representations distributed bilaterally across broad cortical territories under natural speech conditions, provides additional support for the plausibility of a distributed posterior pattern in our data.

The exploratory LCMV projections are consistent with this reframing. These source maps were computed as descriptive anatomical summaries of peak sensor-level effects only and were not used for statistical inference. Because individual structural MRIs were not available and source reconstruction used template anatomy with scalp EEG, the projections should be treated as approximate source-space summaries rather than precise localisations, and anatomical claims should remain limited to broad cortical patterns. Interpreted within these constraints, the source maps support the spoke-level interpretation. Across all three contrasts, the strongest projected desynchronisation was distributed over posterior parieto-occipital and lateral cortical surfaces rather than anterior temporal regions, consistent with differential engagement of sensorimotor and associative spoke networks rather than a hub-level contrast between the two conditions.

### 4.4 Category-sensitive topographies

Our second hypothesis, in which we predicted category-sensitive topographic divergence consistent with the modal grounding of each concept category, was supported. Although the low-beta reduction was common across all three categories, the scalp distributions were not identical. This category sensitivity is important because it suggests that the concept *vs*. dummy contrast captures both a shared semantic retrieval signature and category-dependent differences in the distributed representational network. However, the alignment between the specific predicted patterns and the observed data is variable across categories, and this variability itself provides useful interpretive information.

The Body *vs*. Dummy contrast produced the largest corrected effect and showed a predominantly centro-parietal and posterior distribution (see Table 1, Figures 4, 6). This is partially consistent with the prediction of occipito-parietal and somatosensory involvement. Channels over sensorimotor cortex and parieto-occipital regions were among the strongest contributors, aligning with embodied accounts in which body-related word meanings recruit distributed sensorimotor systems. Hauk et al. (2004) showed that action words referring to different effectors activate motor and premotor regions in a somatotopically organised manner, and grounded accounts more broadly propose that object knowledge is organised according to action, perception, and emotion systems (Martin, 2016). The present body effect should not be interpreted as direct evidence of somatotopic motor activation, because source localisation was exploratory and the strongest scalp effects were centro-parietal and posterior rather than strictly sensorimotor. A more appropriate interpretation is that body concepts elicited a strong low-beta desynchronisation over scalp regions compatible with distributed sensorimotor and posterior association involvement.

The Food *vs*. Dummy contrast showed a broader distribution involving central, frontal, and posterior sites (see Table 1; Figures 7, 9). This is the most consistent pattern relative to the original prediction. Our hypothesis anticipated that deep limbic and subcortical generators would be poorly resolved by scalp EEG and that any observable effects would reflect secondary frontal or insular projections. The observed frontal involvement, including right frontal sites, is broadly compatible with this account. Food knowledge involves visual form, texture, taste, smell, affective value, reward associations, and contextual experience, and many of the regions expected to contribute to these dimensions, including insular, orbitofrontal, limbic, and subcortical structures, are difficult to resolve reliably with scalp EEG. The food contrast should therefore be described as a scalp-visible distributed semantic retrieval effect rather than as direct evidence for specific gustatory or limbic generators. Note that this interpretation remains consistent with grounded and hub-and-spoke accounts, which predict that category-related differences should emerge from the weighting of different modality-specific spokes rather than from entirely separate semantic systems (Lambon Ralph et al., 2017). The exploratory source-space projection observed here should therefore be interpreted as broadly compatible with the food-related meta-analytic pattern reported in our previous ALE study (Radman et al., 2025), while recognising the spatial limitations of scalp EEG.

The Music *vs*. Dummy contrast showed a left centro-parietal and posterior emphasis (see Table 1; Figures 10, 12). This is the least consistent pattern relative to the original prediction. Our hypothesis anticipated a bilateral signature over superior temporal and insular regions reflecting the auditory-motor architecture of music processing. The observed left-lateralised centro-parietal and posterior pattern does not match this prediction. The discrepancy is at least partly explained by the nature of the GRASP clue set. Musical instrument concepts are not purely auditory. They combine object form, action affordances, manual interaction, sound production, cultural knowledge, and auditory imagery. Accordingly, our GRASP clue sentences probed precisely these diffuse associative relations by approaching the target concept from multiple semantic angles rather than cueing sonic properties directly. The left centro-parietal and posterior pattern may therefore reflect retrieval of multimodal instrument knowledge weighted toward object, action, and visual-tactile properties rather than a purely auditory response.

Taken together, the category-specific topographies confirm that the concept *vs*. dummy contrast is sensitive to category-level differences in the distributed semantic network. The alignment with specific fMRI-derived predictions is variable, being strongest for food, partial for body, and weakest for music. This variability reflects both the genuine limitations of scalp EEG spatial resolution and the multimodal nature of clue-based retrieval, which probes distributed associative knowledge rather than isolating any single modality-specific spoke. This graded rather than modular pattern is consistent with a distributed semantic architecture in which semantic retrieval has a shared functional core but different categories and retrieval contexts weight different cortical spokes.

A secondary, relatively ubiquitous, occipital component warrants explicit acknowledgement. Electrode O1 appeared among the top channels in the peak windows for the Body *vs*. Dummy and Food *vs*. Dummy contrasts (Table 1) but not the Music *vs*. Dummy contrast, where the most posterior peak-window involvement was parieto-occipital (see Table 1). However, inspection of the band-by-window theta topographies reveals a more complex picture (see Figures 4, 7, 10). Body shows the strongest and most clearly posterior theta in the sensor maps across the mid to late pre-response windows. Music shows comparably strong posterior theta in the sensor maps, and its exploratory source projection shows the strongest bilateral occipital involvement of the three contrasts (see Figure 12). Food, despite having O1 as a peak channel, shows the weakest posterior theta in the sensor maps and the weakest occipital signal in the source projection (Figure 9).

The pattern is therefore inconsistent across measures and does not map cleanly onto any semantic account based on modal grounding. Body and food share O1 as a peak channel despite having markedly different modal grounding, somatosensory and motor for body and visceral and affective for food, while music lacks O1 as a peak channel yet shows the strongest occipital involvement in the source projection. Differential eye behaviour between concept and dummy trials cannot be excluded as a contributing factor, though note that eye closure typically produces occipital alpha increases rather than the desynchronisation observed here, meaning the directionality does not provide a straightforward ocular account. Critically, the occipital component does not drive the peak frequency selection in any of the three contrasts, which consistently identifies low-beta peaks between 14 and 16 Hz, confirming that it is genuinely secondary to and spectrally dissociable from the primary finding. We do not offer a definitive interpretation of this secondary component. It represents a specific open question for future work combining GRASP with eye-tracking and concurrent neuroimaging to determine whether the occipital involvement reflects a genuine semantic process, a task-related confound, or a methodological limitation of source reconstruction with template anatomy.

### 4.5 Implications for semantic BCI development

Crucially, we believe that our findings are directly relevant to semantic BCI development. A major challenge for semantic BCI is that concept *vs.* concept decoding targets graded, overlapping, and individually variable semantic representations. Previous EEG work has shown that semantic category information can be decoded from single trials, but such decoding remains challenging and is sensitive to design choices. Murphy et al. demonstrated above-chance EEG decoding of semantic category from single concepts, including across participants, but the broader literature still shows that robust cross-participant semantic decoding is difficult (Murphy et al., 2011; Ghazaryan et al., 2023; Rybář et al., 2024). Along these very same lines, Rybář et al. (2024) showed that semantic BCI performance can be substantially overestimated when decoders are allowed to use cue-presentation activity, because cue-locked decoding may capture exogenous sensory responses rather than endogenous semantic processing.

GRASP addresses this problem by shifting the contrast from concept *vs.* concept discrimination to concept *vs.* dummy discrimination and by aligning the analysis to a response-locked marker of concept identification. The complete directional consistency observed across participants, in terms of the low-beta effect, suggests that the presence *vs*. absence of recoverable semantic content may be more participant-invariant than the finer-grained distinction between individual semantic categories. Further, whether this contrast is more participant-invariant than concept *vs*. concept discrimination in a directly matched analysis remains an open question, though the broader literature and our previous classification results (Podmore et al., 2026) suggest this is likely.

Notably, these findings do not indicate that our GRASP paradigm solves semantic decoding. Rather, they provide a clearer signal-characterisation target, specifically that low-beta desynchronisation during the pre-response retrieval period may represent a robust feature for future classifiers. This has two practical implications. First, concept *vs.* dummy decoding may itself be a useful control signal. A system that can detect when a user has entered a high-semantic retrieval state could use that signal to trigger an interface transition, terminate stimulus presentation, or identify the optimal temporal window for subsequent classification. Second, the low-beta effect provides a principled feature space for future machine-learning models. Rather than treating all channels, frequencies, and time points as equally plausible, future sBCI pipelines can prioritise response-preparatory low-beta activity over centro-parietal, posterior, and frontal regions while testing whether category-specific topographic profiles improve classification.

### 4.6 Limitations and future directions

Several limitations should be acknowledged. First, the sample size was modest. However, the consistency of the effect across participants is unusually strong: all 13 participants showed the same negative concept-minus-dummy direction in all three contrasts. This does not remove the need for replication, but it reduces the likelihood that the main effect reflects a small number of influential participants.

Second, the source-space analysis should be interpreted cautiously. The LCMV projections were based on scalp EEG and template anatomy, with no individual structural MRIs captured for reference. As stated in the Methods, these maps were intended as exploratory anatomical summaries of peak sensor-level effects, while statistical inference was based on the whole-scalp TFCE analysis. Future work should combine GRASP with individual MRIs, MEG, or high-density EEG to improve anatomical precision.

Third, the response-locked design is well suited to isolating semantic retrieval and consolidation, but it does not directly characterise word-by-word semantic integration. Because forced-alignment word timings were logged, future analyses could combine our response-locked approach with word-locked analyses to test whether the pseudo-word manipulation produces classical N400-like or time-frequency-based semantic anomaly effects at specific clue words. This would strengthen the link between GRASP and the established ERP literature on semantic processing (Hahne and Jescheniak, 2001; Kutas and Federmeier, 2011).

Fourth, the current results characterise group-level spatio-spectro-temporal effects but do not yet establish real-time decoding performance. The next step is to test whether the low-beta concept *vs.* dummy signature generalises across participants, sessions, and unseen items. A particularly strong test would be cross-category generalisation, training a classifier to distinguish concept from dummy trials in two categories and testing it in the third. Successful cross-category generalisation would indicate that the observed low-beta effect reflects a general semantic-engagement signal rather than a category-specific artefact.

A further consideration concerns the cognitive architecture of the concept *vs.* dummy contrast. The conditions were designed to differ principally in the availability and successful recovery of lexical-semantic content, while retaining sentence-like auditory structure, function words, task engagement, and a matched response requirement. Meaningful concept retrieval is not a unitary or isolated cognitive operation. In the present task, it required the integration of successive clues, lexical and conceptual access, memory retrieval, evidence accumulation, target selection, and the development of sufficient confidence to respond. Dummy trials also recruited active cognitive processing, including anomaly detection, uncertainty monitoring, and the determination that no coherent lexical-semantic interpretation was available. These processes should not, however, be regarded as alternatives to semantic processing. Rather, they form part of the higher-order, distributed cognitive cascade through which meaningful semantic retrieval is achieved, consistent with the distributed hub-and-spoke account of semantic cognition.

We therefore interpret the observed low-beta effect as primarily reflecting differences in neural processing associated with the availability and successful retrieval of meaningful semantic content relative to pseudo-word processing with substantially reduced recoverable lexical-semantic content. The additional response-latency and motor-preparation analyses further indicate that this effect is unlikely to be explained by simple differences in response timing or conventional sensorimotor preparation. The present design does not permit the individual contributions of every linguistic, mnemonic, attentional, integrative, and decisional component of the semantic retrieval cascade to be estimated separately; however, this does not alter the central interpretation of the effect as a robust electrophysiological signature of meaningful semantic retrieval under the present task conditions.

The pseudo-word baseline reflects a deliberate design trade-off. A control condition composed of meaningful but incoherent real words would introduce substantially greater lexical-semantic processing and might encourage continued target search throughout the sentence. The pseudo-word condition instead prioritised a clear reduction in recoverable semantic content while preserving the broad auditory and task structure. Future studies should build upon this paradigm by introducing additional control conditions capable of more finely separating semantic coherence and retrieval from task difficulty, evidence accumulation, decision demands, and confidence.

## 5 Conclusion

Our study provides a response-locked scalp EEG characterisation of meaningful semantic retrieval using the GRASP paradigm. Across body, food, and music concepts, meaningful trials elicited a robust reduction in low-beta power relative to phonologically matched pseudo-word dummy trials. This effect was observed in the pre-response interval, was TFCE-corrected across the full channel, frequency, and time space, and showed complete directional consistency across all participants and all semantic categories. The spatial distribution of the effect varied by category, with body and music showing stronger centro-parietal and posterior involvement and food showing a broader frontal-central-posterior distribution, but all contrasts shared the same dominant low-beta concept-minus-dummy profile.

These findings support the use of pseudo-word sentences as an active low-semantic reference condition for isolating semantic retrieval in EEG. The first hypothesis, predicting bilateral anterior temporal hub engagement, was not confirmed at the scalp level. This is likely owing to the dummy condition reducing rather than eliminating semantic processing, meaning both conditions likely engage the bilateral ATL hub to some degree. The second hypothesis, predicting category-sensitive topographic divergence reflecting modal grounding, was partially supported, with distinct scalp distributions observed across the three categories pointing toward differential spoke engagement. The results should not be overinterpreted as precise anatomical localisation, because source projections were exploratory and based on template anatomy.

For semantic BCI development, the key contribution is the identification of a stable and participant-invariant electrophysiological contrast between meaningful and low-semantic processing. Rather than beginning with the more difficult problem of decoding which concept is being retrieved, GRASP identifies a reliable neural signature of whether meaningful retrieval is occurring at all. This low-beta concept *vs*. dummy effect provides a principled target for future decoding, cross-participant generalisation, and closed-loop semantic BCI development.

## Data Availability

The datasets presented in this article are not readily available because the data are currently restricted as the authors are preparing a separate open-source repository paper for publication following the prospective acceptance of this article. Requests to access the datasets should be directed to mr24965@essex.ac.uk.
