# Supplementary material for: GRASP: a response-locked paradigm that reveals low-beta desynchronisation is a participant-invariant signature of semantic decoding in EEG
Source: Front Hum Neurosci. 2026 Sep 14;20:1913105. doi: 10.3389/fnhum.2026.1913105 (PMC13617028; doi:10.3389/fnhum.2026.1913105)
Supplement: Supplementary file 1 [file Data_Sheet_1.docx]

**Table S1. Behavioural performance before EEG-based and epoch-level behavioural filtering**

| **Measure** | **Overall** | **Body** | **Food** | **Music** |
| --- | --- | --- | --- | --- |
| Concept exact-match accuracy, % | 79.50 (7.50) | 85.27 (5.91) | 81.95 (9.80) | 71.32 (8.78) |
| Mean cosine similarity, % | 89.00 (4.50) | 90.17 (3.96) | 91.02 (5.55) | 85.72 (4.92) |
| Concept RT mean, s | 6.28 (0.48) | 6.26 (0.53) | 5.85 (0.50) | 6.81 (0.45) |
| Concept RT median, s | 6.33 (0.51) | 6.26 (0.54) | 5.90 (0.53) | 6.93 (0.43) |
| Dummy correct, % | 100.00 (0.00) | 100.00 | 100.00 | 100.00 |
| Dummy RT mean, s | 2.84 (0.51) | 2.69 (0.46) | 2.87 (0.59) | 2.96 (0.53) |
| Dummy RT median, s | 2.62 (0.51) | 2.46 (0.42) | 2.75 (0.69) | 2.72 (0.46) |

Table 1. Values represent participant-level means, with between-participant standard deviations in parentheses. Concept accuracy is the exact-match rate between the participant’s typed response and the target item label. Mean cosine similarity was calculated between the word2vec embeddings of the SymSpell-corrected response and the corresponding target label. Response latency was measured from trial-audio onset to the spacebar press and was calculated only for trials in which a spacebar response occurred. Category-specific dummy results correspond to pseudo-word sentences derived from clues belonging to the respective semantic category. Trial-by-trial confidence ratings were not collected.

**Figure S1. Channel-level relationship between response latency and low-beta power. Topographic distribution of Pearson correlation coefficients between response latency and mean log10-transformed low-beta power in the 13–20 Hz band, calculated independently at each of the 64 EEG channels using the same 3,408 retained epochs. Ten channels survived Bonferroni correction across the 64 comparisons** $\left( \boldsymbol{\alpha=0.05}\mathbf{/}\boldsymbol{64=7.8\times}\boldsymbol{10}^{\boldsymbol{-4}} \right)$**: Iz, F8, F4, P9, P6, P4, PO4, P8, P2, and PO8. However, the corresponding effect sizes were small, with response latency explaining only 0.35–0.74% of the variance in low-beta power. The surviving associations differed in direction across the scalp, were not concentrated over the conventional sensorimotor region, and showed only limited overlap with the highest-ranked electrodes contributing to the principal concept-versus-dummy effects.**

**Figure S2. Pre-response sensorimotor mu activity for concept and dummy trials. Grand-average mu-band power in the 8–13 Hz range for concept and dummy trials at the standard sensorimotor channels C3, C4, and Cz across the −1.5 to 0 s response-locked interval. For each participant, power was expressed relative to the participant’s own −1.5 to −1.3 s reference window using a log-ratio transformation, before averaging across participants. Shaded regions indicate ±1 standard error of the mean. Both conditions showed overlapping pre-response mu desynchronisation, and no time interval at any of the three channels reached the cluster-corrected significance threshold in the sign-flip permutation analysis with 2,000 permutations.**
